# The uulmMAC Database—A Multimodal Affective Corpus for Affective Computing in Human-Computer Interaction

**DOI:** 10.3390/s20082308

**Published:** 2020-04-17

**Authors:** Dilana Hazer-Rau, Sascha Meudt, Andreas Daucher, Jennifer Spohrs, Holger Hoffmann, Friedhelm Schwenker, Harald C. Traue

**Affiliations:** 1 Section Medical Psychology, University of Ulm, Frauensteige 6, 89075 Ulm, Germany; 2 Institute of Neural Information Processing, University of Ulm, James-Frank-Ring, 89081 Ulm, Germany

**Keywords:** affective corpus, multimodal sensors, overload, underload, interest, frustration, cognitive load, emotion recognition, stress research, affective computing, machine learning, human-computer interaction

## Abstract

In this paper, we present a multimodal dataset for affective computing research acquired in a human-computer interaction (HCI) setting. An experimental mobile and interactive scenario was designed and implemented based on a gamified generic paradigm for the induction of dialog-based HCI relevant emotional and cognitive load states. It consists of six experimental sequences, inducing *Interest, Overload, Normal, Easy, Underload*, and *Frustration*. Each sequence is followed by subjective feedbacks to validate the induction, a respiration baseline to level off the physiological reactions, and a summary of results. Further, prior to the experiment, three questionnaires related to emotion regulation (ERQ), emotional control (TEIQue-SF), and personality traits (TIPI) were collected from each subject to evaluate the stability of the induction paradigm. Based on this HCI scenario, the *University of Ulm Multimodal Affective Corpus (uulmMAC)*, consisting of two homogenous samples of 60 participants and 100 recording sessions was generated. We recorded 16 sensor modalities including 4 × video, 3 × audio, and 7 × biophysiological, depth, and pose streams. Further, additional labels and annotations were also collected. After recording, all data were post-processed and checked for technical and signal quality, resulting in the final *uulmMAC* dataset of 57 subjects and 95 recording sessions. The evaluation of the reported subjective feedbacks shows significant differences between the sequences, well consistent with the induced states, and the analysis of the questionnaires shows stable results. In summary, our *uulmMAC* database is a valuable contribution for the field of affective computing and multimodal data analysis: Acquired in a mobile interactive scenario close to real HCI, it consists of a large number of subjects and allows transtemporal investigations. Validated via subjective feedbacks and checked for quality issues, it can be used for affective computing and machine learning applications.

## 1. Introduction

The rapid technological advancements and the expectations for fast adaptation impose high pressure on humans to deliver maximum effort in stressful constraints and multitasking situations of HCI. Among the variety of emotional and cognitive states in HCI, cognitive load is a prominent “multi-dimensional construct representing the load imposed on the working memory during performance of a cognitive task” [1]. It is highly associated with human effort and with the efficiency of cognitive technical systems during Human-Computer Interaction (HCI) [2]. Following Sweller [3], which focuses on human learning, the intensity of cognitive load experienced for a specific mental task varies between individuals depending on their working memory capacity. Individuals can raise their cognitive effort to adapt to increasing difficulties until mental limit capacities are reached. Above this limit, human performance decreases, involving increase in errors, emergence of stress, and negative affects [2]. An adequate level of cognitive load for an individual is desirable, in order to perform a task in an optimal manner. Results from our transsituational study show indeed the existence of a biological basis for success in human-computer interaction [4]. Therefore, particularly in the context of HCI, knowledge about cognitive load is essential in order to intelligently match the level and nature of the interaction in such systems. The recognition of cognitive load in HCI can enable real-time user’s state monitoring and adaptation to the individual users. Individual content generation for distant learning and adaptive learning systems [5], practical training sessions [6], monitoring pilots [7], and truck drivers [8], usability testing and evaluation of user-interface and mobile applications [9], or digital assistance providing personalized advises for stress reduction and health risk prevention strategies [10] are some relevant fields of useful applications. 

Estimation of cognitive load can be achieved via various measuring approaches, including subjective measures, performance measures, physiological measures, and behavioral measures [11,12,13,14,15]. Traditional simple measures are based on subjective ratings, asking the users to perform a self-assessment of their mental state. These measures lack objectivity and are not reliable for computational recognition techniques. They are generally used as ground truths in experiments, however with the disadvantage of being acquired after the event. Performance measures can be measured in parallel, but are difficult to evaluate in real-life applications and generally insensitive to load capacity variations. Physiological and behavioral procedures are non-intrusive methods providing a more reliable and direct access to cognitive load in an objective way. Cognitive load recognition using multimodal sensors has the potential to increase the robustness and accuracy compared to estimation from single modality data. Unlike subjective measurements prevalent in psychological research, cognitive load estimation based on human responses is necessary for advanced computational techniques. Further, real-life investigation requires the implementation of mobile measurements “in-the-wild” [16]. Despite all technological advancements, mobile measurements still represent a challenge and can only be realistic if the measuring devices and sensor techniques are reliable and sensitive to wild movements, are at low cost, and easy to wear. 

Various datasets were specifically collected for the study of cognitive load. While most of the studies are based on statistical approaches or functional magnetic resonance imaging (fMRI) [17,18], alternative methods including physiological [19,20], text [21,22] speech [23,24], brain [25,26], and pupil change [27,28] analyses, are used to detect cognitive load. The relationship between cognitive load and writing behavior was examined using the CLTex (Cognitive Load via Text), CLSkt (Cognitive Load Sketching) and CLDgt (Cognitive Load via Digits) datasets [29]. The datasets are composed of writing samples of 20 subjects under three cognitive load levels, induced from a writing task experiment. Speech-based cognitive load examination is supported by the Cognitive Load with Speech and Electroglottography (CLSE) dataset [30]. It includes recordings of 26 subjects for the determination of a speaker’s cognitive load during speech based on acoustic features. Mattys et al. developed an experiment to induce cognitive load based on a concurrent visual search task for the investigation of the impact of cognitive load on the Ganong effect [24]. The effect of visual presentation was also investigated for the detection of cognitive load: Liu et al. present a contact-free method to improve cognitive load recognition from eye movement signals and for this purpose designed an experiment to induce cognitive load [31]. In their final project report for AOARD Grant, Chen et al. summarize research activities and issues related to multimodal cognitive load recognition in the real world. They examine the use of various electroencephalography (EEG) features, eye activities, linguistic features, skin conductance response, facial activities and writing behavior. An extended version of the report is their book “Robust multimodal cognitive load measurement” presenting all the related issues in details [29]. 

As for the induction of emotional states, many studies exist focusing on basic emotions in both discrete (i.e., fear, anger, joy, sadness, surprise or disgust) or dimensional (i.e., valence, arousal, dominance) models. These emotional states are especially induced using standardized pictures [32,33] for instance from the International Affective Picture System (IAPS) [34] or relying on audiovisual stimuli [35] used as movie clips [36,37] or as music clips [38]. Emotional states can be also induced using game scenarios by asking the user to perform a certain task [39]. This elicitation method is especially useful for the induction of HCI relevant emotional states such as *Frustration* and *Interest* [40]. These states are relevant in designing efficient and easy-to-use interactive systems [41], in interactive educational and social applications [42], or in therapeutic settings by providing tailored feedback for instance to reduce *Frustration* states [43].

Taylor et al. conducted a study to induce *Frustration* in subjects based on the inclusion of latency between the user’s touch and the reaction of the breakout engine [44]. A more recent study on *Frustration* is given by Aslam et al. examining the effects of annoying factors in HCI on feelings of *Frustration* and disappointment [45]. For the induction of *Frustration*, they asked the subjects to fill in a registration form, which fails twice based on intended system errors, before it succeeds in the third time. Additionally, Lisetti et al. designed an experiment for the elicitation of six emotions including *Frustration* in the context of HCI [46]. They collected physiological data via wearable computers and included classification results of three different supervised learning algorithms. In their paper on human-robot interaction, Liu et al. present a comparative study of four machine learning methods using physiological signals for the recognition of five different emotions including *Frustration* [47].

In her article “Interest—the curious emotion”, Silvia focuses on the role of *Interest* in learning and motivation and describes its central role in cultivating knowledge and expertise [48]. Additionally, Reeve et al. present a concept of *Interest* in three ways: as a basic emotion, as an affect, and as an emotion schema [49]. They explain the importance of *Interest* in educational settings as a mean to motivate high-quality engagement that leads to positive learning outcomes and as an enrichment of motivational and cognitive resources that leads to high-vitality experience rather than exhaustion. According to Ellsworth, *Interest* can be related to the uncertainty of a positive event which may also lead to curiosity and hope, while lack of control often results in *Frustration*, which if sustained can lead to desperation and resignation [50]. Thus, in a HCI context, providing excitement through an appropriate degree of uncertainty might increase *Interest*, while providing a certain level of controllability, by preventing inexplicit system errors can reduce *Frustration*. The recognition of *Frustration* and the system reaction to turn it into a positive *Interest* state are critical aspects for avoiding negative affective consequences and valuable for enhancing positive interaction effects. 

Despite the many studies investigating emotional and cognitive states, particularly *Overload*, *Underload, Frustration* and *Interest*, their measurement still poses many challenging issues especially with respect to multimodal, mobile and transtemporal acquisition. Additionally, regarding the validation of the experimental induction, most of the studies limit their validation to one subjective modality. Further, previous studies restrict their induction to either cognitive or emotional elicitation and rarely include both states into one single dataset. In this paper, we focus on these issues and present a database for affective computing research, based on systematic induction of cognitive load (*Overload*, *Underload*) and specific emotions relevant to HCI (*Interest*, *Frustration*) as well as a neutral and a transition state (*Normal, Easy*) (see Section 2.2). The database is (1) designed and acquired in a mobile interactive HCI setting, (2) based on multimodal sensor data, (3) involving transtemporal acquisition including different recording times, and (4) validated via three different subjective modalities. Combining these challenging issues related to mobile, interactive, multimodal, transtemporal, and validated acquisition into one large dataset for both cognitive and emotional states are the main contributions of this work. 

In the next section (Section 2), the methods are described including a description of the participants and cohorts, interaction scheme, experiment structure, technical implementation and multimodal sensors infrastructure. Following (Section 3), the results are presented including the generated *uulmMAC* database, the validation via questionnaires and subjective feedback, as well as the data annotation. Finally (Section 4), we conclude with a discussion and a summary of the results.

## 2. Materials and Methods

An experimental mobile interactive and multimodal emotional-cognitive load scenario was designed and implemented for the induction of various cognitive and emotional states in an HCI setting. Based on this mobile and interactive scenario, multimodal data were acquired generating the *University of Ulm Multimodal Affective Corpus (uulmMAC)*. The basic concept of our cognitive load scenario follows a generic scheme from Schüssel et al. who proposed a gamified setup for the exploration of various aspects with potential influence on users’ way of interaction [51]. The generic scheme is, however, an abstract fundament for HCI exploration with no specific application field. The induction of emotional and cognitive states depends on various factors related to the specific nature of human reactions [52]. Therefore, for our research question focusing on emotional and cognitive states induction in real-life HCI, the development of the current experiment required further developments with an in-depth adjustment and re-implementation of the original generic paradigm such that to comply with the induction requirements of cognitive load and affective states. The main development contributions include the design of the interaction sequences scheme inducing cognitive, emotional and neutral states (Section 2.2), the development of the experimental structure (Section 2.3) and the software implementation and platform embedment (Section 2.4). Furthermore, for the experimental data acquisition, we developed and implemented a technical infrastructure with multimodal sensors system for the distributed experimental and recording setup (Section 2.5).

### 2.1. Participants and Cohort Description

The *uulmMAC* dataset consists of two homogenous samples of 60 participants (30 females, 30 males; 17–27 years; mean age = 21.65 years, SD = 2.65) with a total of 100 recording sessions (N = 100) of about 45 minutes each. The 60 subjects are medical students and were recruited through bulletin notices distributed at the campus of the Ulm University. The first sample includes 40 subjects who underwent one measurement each, while the second sample consists of 20 subjects who underwent three measurements each. The three different measurements were acquired at three different times with one week of time-interval in-between. The second sample allows for instance the investigation of additional transtemporal research questions. While both samples underwent exactly the same experiment, they slightly differ in one modality acquisition: The first sample does not include facial electromyography (EMG) measurements, allowing better conditions for the analysis of facial expressions via video data. Both samples are evenly balanced between male and female. All subjects gave their informed consent for inclusion before they participated in the experiment and the study was approved by the Ethics Committee of the Ulm University (Project: C4 - SFB TRR62).

In summary, the original dataset of *uulmMAC* consists of 100 individual recording sessions: The first sample with 40 recording sessions (40 subjects × 1 measurement) and the second sample with 60 recording sessions (20 subjects × 3 measurements). 

### 2.2. The Interaction Scheme

**Figure 1:**
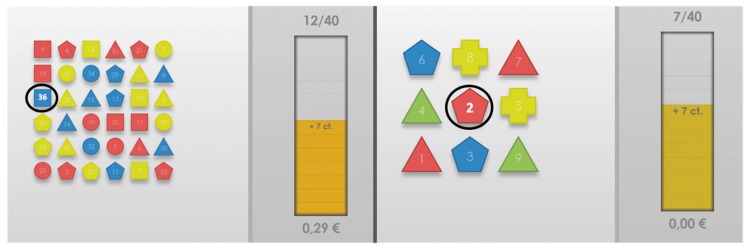
The visual search task on the example of *Overload* (**left**) and *Underload* (**right**) induction scheme. The user has to spot the single unique object. The correct answers are 36 for *Overload* (unique blue and square object) and 2 for *Underload* (unique red and pentagon object).

The goal of the experiment was the induction of various dialog-based cognitive and emotional states in a real HCI environment. Therefore, the participants were asked by the system to solve a series of cognitive games in order to investigate their reaction to various cognitive tasks difficulties, varying from high interest and overwhelming to boring and frustrating levels. The aim of each game task was to identify the single one item that is unique in shape and color (i.e., the number 36 and the number 2 in Figure 1), based on a visual search task. The difficulty was set by adjusting the number of objects, shapes and colors shown per task as well as the available time given to solve that task. Thus, cognitive *Overload* was induced by increasing the task field objects and decreasing the available time, while cognitive *Underload* was induced by decreasing the task field objects and increasing the available time. Further, for each individual task, the subject could earn a certain amount of money (up to ten cents) according to the individual speed of the given response. The amount of reward money earned for solving a task was increasingly reduced, the longer the subject needed to answer. If the given answer was incorrect, the participant received no reward at all for that particular task. Figure 1 shows screenshots of the visual search task.

### 2.3. Experiment Structure

The experiment structure consists of six *induction sequences*, separated by *subjective feedback* related to the actual sequence, and followed by a respiration *baseline* and a *summary* of the achieved results in that sequence. While the experimental sequences are used to induce various cognitive and emotional states, the subjective feedback is used for the validation of the induction. These are described in details in the following subsections. Further, prior to the experiment, each subject received an introduction and instructions to the experimental steps in form of a short PowerPoint presentation and was afterwards asked to fill in three questionnaires related to: (1) emotion regulation based on the Emotion Regulation Questionnaire (ERQ) [53,54]; (2) emotional control based on the Trait Emotional Intelligence Questionnaire Short Form (TEIQue-SF) [55,56]; and (3) personality traits based on the Ten Item Personality Measure (TIPI) [57,58]. These questionnaires are also used as further subjective evaluation of the stability of the induction paradigm.

#### 2.3.1. Induction Sequences

Six consecutive sequences of different difficulties, with 40 single tasks each, are implemented for the induction of six different emotional and cognitive load states. All tasks within a sequence have thereby the same or comparable difficulty levels. The first introductory sequence is designed to induce *Interest* and is of moderate difficulty to gain the users’ interest and familiarize them with the visual search task procedure. The *Interest* sequence has 40 tasks and is designed with a mix of 3 × 3 and 4 × 4 matrices, and 10 s time per task to give the right answer. The second sequence is designed to induce *Overload* and consists of 40 difficult tasks with a 6 × 6 matrix each and with short time of 6 s per task to provide an answer. The third sequence has a moderate *Normal* difficulty and is defined with 40 tasks with 4 × 4 matrices and moderate time of 10 s to respond per task. This *Normal* sequence is the neutral (cognitive and emotional) state to be considered as baseline between the sequences. The fourth sequence is implemented as an *Easy* sequence with 40 tasks with 3 × 3 matrices and very long time of 100 s for responding. In order to induce *Underload*, the fifth sequence is defined as a repetition of the previous *Easy* scheme of low difficulty, with again 40 tasks with 3 × 3 matrices and 100 s to provide an answer. This originates from the trivial idea that repeating an easy well-known task in the same way two times in a row, generates a state of boredom and leads to *Underload*. Based on this idea, the *Easy* sequence is considered as a transition state used as a mean to induce *Underload*. Finally, the last sixth sequence is intended to induce *Frustration* by purposely logging in a wrong answer at randomly distributed tasks (eight wrong out of 40), even when the subject provides a right answer. This *Frustration* sequence has 40 tasks with a mix of 3 × 3 and 4 × 4 matrices each and 10 s time to provide an answer. Table 1 illustrates a summary of the experimental procedure. 

The user-system interaction during all the tasks is a mobile interaction conducted via natural speech while the participants could freely move and walk in the room (standing position). The walking area is limited to a field of 1 m × 3 m, represented by an electrostatic floor mat to prevent any signal disturbance caused by any electrostatic charge influence.

#### 2.3.2. Subjective Feedback

**Figure 2:**
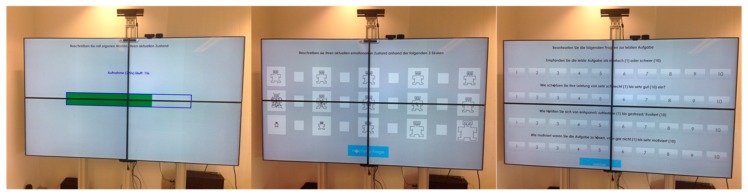
Illustration of the subjective feedback screens including Free Speech (**left**), SAM Ratings (**middle**) and Direct Questions (**right**) parts. Note that the SAM Ratings are scored on a nine-point Likert scale (represented by both the big labeled fields and the small empty fields).

In order to evaluate the validity of the induction paradigm, various kinds of subjective feedback are implemented, including *Free Speech*, *SAM Ratings*, and *Direct Questions* parts. These are presented to the subjects on the screen as illustrated in Figure 2. After each of the six accomplished sequences, the participants provided a series of information about their current emotional state in three different ways, including: (1) expressing in own words via *Free Speech* feedback of 12 s duration, how they felt during that particular sequence, (2) rating their emotions via Self-Assessment-Manikin *SAM Ratings* on the Valence-Arousal-Dominance (VAD) scale, and (3) answering *Direct Questions* related to the assessment of their own performance. The aim of this subjective feedback is to determine the current subjective emotional state experienced in that particular sequence, which, in turn, can be used as ground truth to evaluate and validate the induction paradigm. While the Free Speech feedbacks are given via natural speech, logging of the SAM Ratings and Direct Questions was carried out per mouse-click to ensure correct logging documentation. The user was thereby guided and instructed by the system via speech output. The user-system interaction modality (mouse, speech or both) within the experiment is part of the technical implementation as described in Section 2.4.

#### 2.3.3. Respiration Baseline and Results’ Summary

Following the subjective feedback, a baseline phase consisting of a breathing exercise to level off the physiological reactions related to that particular sequence is conducted by the subjects. Additionally, here, the users are thereby guided by the system via speech to first deeply breathe, then hold their breath for few seconds, and finally breathe out. The exercise was repeated three times subsequently. Finally, after the baseline phase, the system informs the user via speech about his performance during the last sequence and the related results achieved, including the earned money, are presented on the screen.

### 2.4. Technical Implementation

The further developments of the generic paradigm and software implementation of the interaction scheme and experimental structure for the induction of various cognitive, stress, and affective states are realized using C# programming and integrated within the Semaine platform [59].

The workflow of the experiment including the structure, order and content of the different sequences as well as the subjective feedback and baseline sections in between are defined in an external *taskset* file which can be imported at the beginning of the experiment. Within a *taskset*, the course setting of the sequences can be defined individually for every task and every subject, allowing a high flexibility and an easy-to-handle workflow setup. Additionally, the user-system interaction modality (mouse, speech, or both) for every part within the experiment is predefined in this file. This also includes the text content (spoken and written) given by the system. The *taskset* describes the course of events of the entire experiment and is consistent for all the participants, except for the second sample who underwent three repeated measurements at three different times. For this group, the content of speech output given by the system is slightly modified for the second and third measurements by using alternative synonyms while keeping the content the same. The intention here is to keep the interaction as natural as possible by preventing a repetition of exactly the same words every time. 

During the visual search task, the user is instructed to give his answer by speech command. To recognize the speech content, our experimental implementation includes an integrated automated speech recognition algorithm. If well trained in advance, the speech recognition works properly in most of the cases. Nevertheless, in order to ensure a smooth interaction between the user and the system, a “Wizard of Oz” (WOZ) scenario was also implemented and used to support the integrated automated speech recognition algorithm. This was especially useful if the automated recognition fails for instance because of language dialect disparity of specific subjects that strongly diverge from the norm language on which the recognition algorithm was trained. Within the WOZ scenario, the experiment was observed on an external monitor in a separate room by the experimenter, who controlled and adjusted the (correct) login of the given answers, if necessary.

Finally, the behavior of the subjects and all their conducted actions as well as the whole course of the experiment are triggered after the events. As a result, for every individual subject, a .log file is generated after every experiment including all the course details of the experiment and can be used for the later processing and analysis of the signal data.

### 2.5. Multimodal Sensors for Data Acquisition

In order to collect high quality data for a wide kind of multimodal analysis there are mainly two important issues regarding the technical data acquisition. First, a wide set of different modalities with a maximum of data quality in each sensor needs to be ensured. Second, the synchronization between all sensors and the user interface components has to be as congruent as possible. The sensors used here can be divided into two kinds. Sensors attached to the participant and sensors mounted to the environment. To ensure a high mobility of the participant, and, therefore, less influence on the participant’s natural behavior, wireless sensors were used. 

In particular, they include a small theatre stereo headset microphone with a frequency range of 20 to 20.000 Hz, sampled at 48 kHz, transmitted via digital radio and a g.tec g.MOBIlab+ Bluetooth amplifier for biophysiological sensors. The bioamplifier was equipped with sensors for electromyography (EMG), electrocardiography (ECG), skin conductance level (SCL), respiration, and body temperature at a sampling rate of 256 Hz. To ensure accurate recordings free of motion artifacts, the signals from the physiological sensors underwent an online monitoring check adapted for our experiment using Simulink® software. This online signal quality check was conducted during an initial baseline record at rest in sitting position and prior to the first sequence of the experiment. 

**Figure 3:**
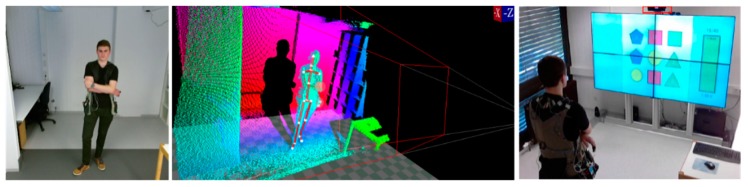
View of the frontal camera (**left**), depth information (**middle**) and scenery overview of the rear camera (**right**).

A stationary mounted frontal webcam with HD resolution of 1920 × 1080 pixels at 30 frames per second was used. Further a Microsoft Kinect v2 also was mounted in the front. The Kinect includes a full HD RGB color video stream (1080p @ 30 Hz), an infrared (IR) video stream (512 × 424 @ 30 Hz), a depth stream (512 × 424 @ 30 Hz), a directed audio stream (virtual beam forming by a microphone array) and pose estimation stream including skeleton information containing 25 joints. Kinect and primary webcam were placed on top of the interaction screen in front of the scenery looking towards the participants face. Finally, a second webcam with a resolution of 1280 × 720 @ 30 fps was placed in the rear of the experimental setting in order to monitor the scenery overview and sample the atmosphere sounds. Figure 3 shows the views from the frontal and rear cameras and the acquired depth information.

Summarized, we recorded 16 sensor modalities, including four video streams (front/rear/Kinect RGB/Kinect IR), three audio streams (headset/directed array/atmosphere), seven biophysiological streams (3 × EMG/ECG/SCL/respiration/temperature), depth, and pose stream. Further, several label information streams extracted from an application log file, described later, were also recorded. After recording, all data were post-processed in order to prove a high quality towards technical and signal quality issues. As visualization tool we used ATLAS [60,61] to present (and playback) all recorded data to the experts. Only sessions which passed all technical and manual quality checks belong to the final dataset of 100 (40 × 1 + 20 × 3) sessions. These are described in Section 3. In addition to the annotation extracted from the log file entries and experimental design structural issues, some additional labels are achieved by a semi-automatic active learning procedure as described in the Annotation section (Section 3.4) of this work. 

**Figure 4:**
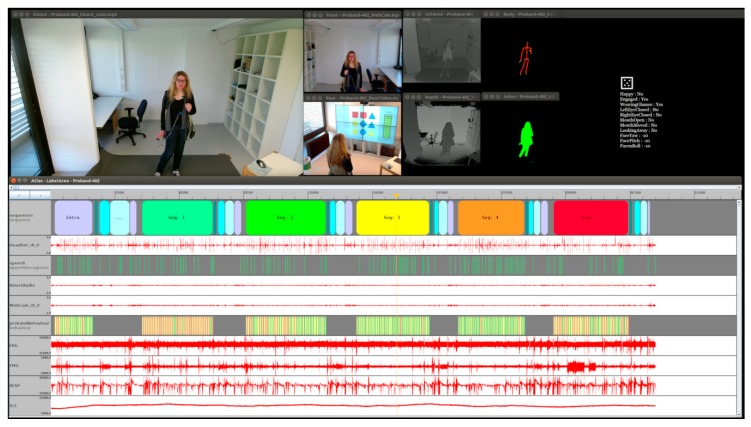
Overview of a whole recording session displayed in the multimodal annotation tool ATLAS: Kinect video (**top left**); Front webcam view, infrared and pose (first video row); rear camera, depth images (second video row); face position and simple facial estimations (**top right**). The data window contains from top to bottom: Sequence start and end information from logfile, audio, speech recognition information from logfile, stereo audio, front webcam, search, and answer phases including hit and miss from logfile, ECG, EMG, respiration, SCL.

Figure 4 shows an overview of the collected data of a single session displayed in the visualization tool ATLAS. All video streams, time series type data and some label information are illustrated. The timescale is at minimum zoom, so the structure of experimental phases can be seen in the upper annotation line. It is not possible to record this massive amount of data on a single PC, so we developed a modular network-based recording infrastructure called MAR^2^S (Multimodal Activity Recognition and Recording System). This contains a specific recording module which on the one hand controls each specific sensor according to its specific API. This can include preparation and initialization commands, trigger and timing control, data format transformations, disk read/write control of the streams, etc. On the other hand, each module accomplishes the defined network commands and synchronization protocols. The modules are mostly written in C#, but due to the inter-module communication by network, there is no technical limitation to a specific programming language, operating system or hardware type. Depending on the sensors, hard and software requirements, in most cases more than one sensor can be grouped on a PC without influencing each other.

In addition to the sensor modules, the user interface (UI) and WOZ module were also encapsulated in such a network module in order to control and monitor their behavior in the same synchronous manner. Finally, a logging module was established acting like a sensor, not recording physical data, but recording the whole system behavior. This includes exact time stamps on all participants and WOZ inputs, global information on the internal and external systems states, information about the sensors states, any network communication, etc. With this log file and the recorded sensor streams it is possible to reconstruct the whole experimental procedure in detail up to a virtual playback without a real participant. Therefore, the data can not only be used for numerous offline analyses but also for the development of real time capable online recognition systems.

**Figure 5:**
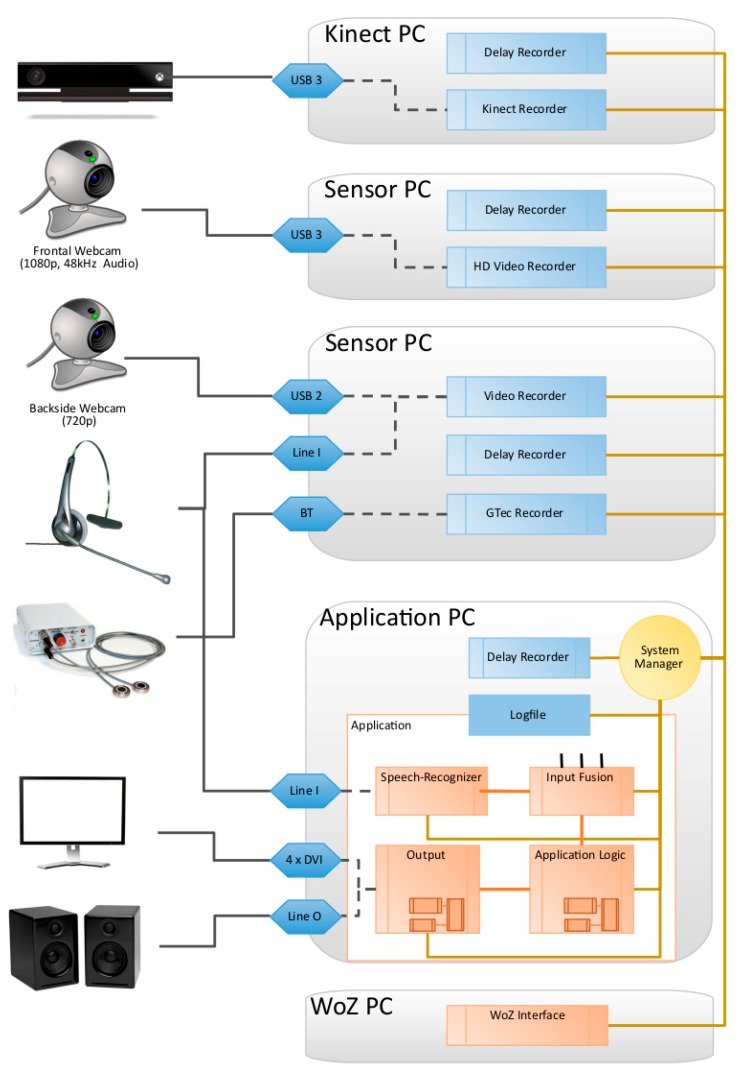
Technical infrastructure of the distributed experimental and recording setup.

Finally, each involved PC had a network monitoring module, measuring the current network latency to ensure synchronous recording. Due to the usage of “of the shelf” sensors like the Kinect sensor and webcams, which do not include physical trigger input capabilities, and the complex multi-PC network environment, we are not able to ensure synchronicity on a nanosecond level, like highly-specialized, expensive, hardware-triggered setups do. Hence, our setup is much more flexible and a great deal more realistic towards future end user implementations on custom hardware and smart devices. The recorded emotions and mental states occur typically in a longer range and all multimodal recognition approaches typically use time windows from 50 ms up to several seconds. Thus, inter-modality delays from under one millisecond are acceptable. To ensure this, each involved PC was directly attached to a separate recording control sub network containing just one switch transmitting only record timing and control information (no sensor streams, they are processed locally). Figure 5 shows the technical infrastructure of the distributed experimental and recording setup.

The module which initiates the recording start also listens to its own send “start” message, and starts recording after the message returns back to itself to prevent time leading of the initiator module. Each module further sends a roundtrip message to itself to measure the network latency at the beginning of each recording session. The round trip times can be seen in Table 2. Thus, we can assume that the average delay or desynchronization is within an acceptable range. Additionally, the synchronicity can be improved by taking the individual delays into account and shifting the timestamps after recording in the post-processing step. This is not done in the raw data. 

## 3. Results

In the following, the resulting database, the validation of the induction via questionnaires and via subjective feedback, as well as the data annotation results are presented.

### 3.1. The Database

In total, three subjects were excluded from the analysis: two subjects from the first sample because of missing biosignal data (ID-04) and an absent logger data (ID-40) as well as one subject from the second sample because of missing sequences due to a technical error (*Underload* and *Frustration* for ID-90). Because ID-90 represents the second measurement of a participant from the second sample, the first and third measurement data of that subject (ID-80 and ID-100) were also excluded from the analysis. Consequently, the final dataset *uulmMAC* consists of 95 recording sessions from 57 subjects, presented for the following groups and subgroups:

- Group A involves 38 subjects from the first sample who underwent one single measurement. It consists of 38 recording sessions.
- Group B involves 19 subjects from the second sample who underwent three different measurements. It consists of 57 recording sessions. Group B includes three different subgroups: Group B1, Group B2, and Group B3 consisting of 19 recording sessions each and representing the first, second and third measurement time of the 19 subjects, respectively.

While both groups underwent exactly the same experiment, they slightly differ in one modality acquisition: The EMG data of Group A include only musculus trapezius activity measurements (thus, without facial electrodes, which allows a better analysis of facial expressions from the video data). As for Group B, the EMG data include activity measurements of three muscles: musculus trapezius, musculus currogator and musculus cygomaticus. In the following, the results of Group A, Group B1, Group B2, and Group B3 are separately analyzed and presented.

### 3.2. Evaluation via Questionnaires

The three questionnaires TEIQue-SF, ERQ, and TIPI collected from all the participants prior to the experiment are first evaluated for Group A, Group B1, Group B2, and Group B3. For all questionnaires items, the possible score values range between 1 (minimum) and 7 (maximum).

#### 3.2.1. TEIQue-SF Questionnaire

**Figure 6:**
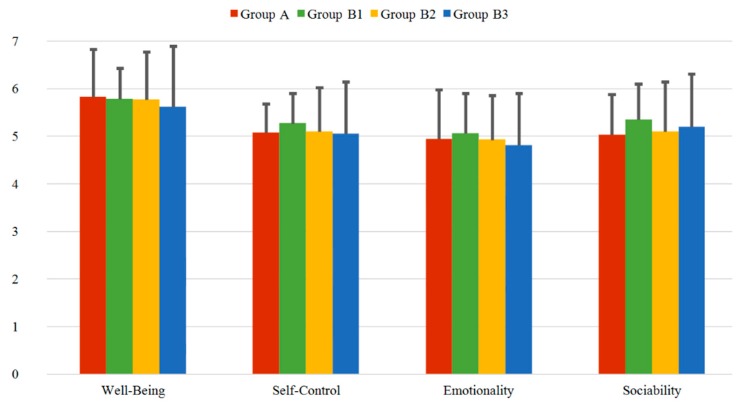
Mean values of the four dimensions of the TEIQue-SF questionnaire for the different groups. The error bars represent the corresponding standard deviations.

In Figure 6 the four dimensions of the TEIQue-SF, consisting of Well-Being, Self-Control, Emotionality, and Sociability factors, are presented for the different groups. The mean values vary between 5.61 and 5.82 for the Well-Being factor, between 5.04 and 5.26 for the Self-Control factor, between 4.80 and 5.06 for the Emotionality factor and between 5.02 and 5.34 for the Sociability factor. The standard deviations (SD) range between 0.55 and 1.28 for all groups and all factors. The first three factors of Well-Being, Self-Control and Emotionality have a small decreasing tendency within Group B1, Group B2 and Group B3, with the highest value obtained for Group B1. Only for Sociability the mean value of Group B3 slightly increases compared to the value of Group B2. Nevertheless, in total the mean values of all factors present a homogenous distribution within all the groups, showing minimal deviations and, thus, stable results. 

#### 3.2.2. ERQ and TIPI Questionnaires

**Figure 7:**
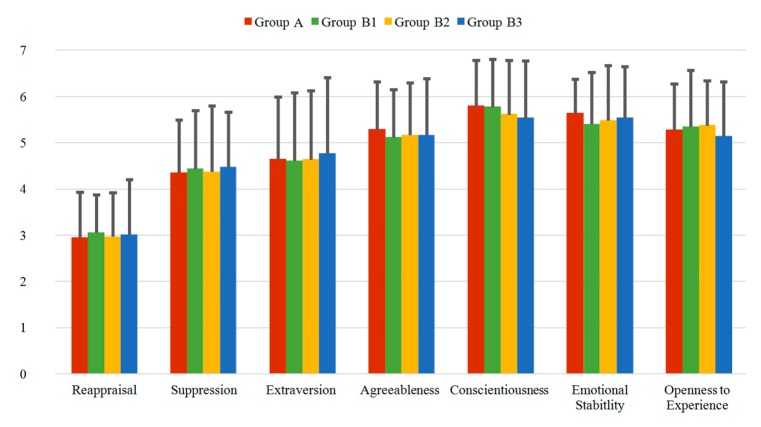
Mean values of the ERQ and TIPI questionnaires for the different groups: Reappraisal and Suppression are the ERQ dimensions, while Extraversion, Agreeableness, Conscientiousness, Emotional Stability, and Openness to Experience are the TIPI dimensions.

In Figure 7 the results of the ERQ and TIPI questionnaires are presented for Group A, Group B1, Group B2 and Group B3. Reappraisal and Suppression are the factors related to the ERQ, while Extraversion, Agreeableness, Conscientiousness, Emotional Stability and Openness to Experience are the factors related to the TIPI questionnaire. The range of the Reappraisal mean values varies between 2.94 and 3.05 (range of SD: 0.81 to 1.19), while the range of the Suppression values varies between 4.35 and 4.46 (range of SD: 1.14 to 1.43). For the TIPI questionnaire, the Extraversion has values between 4.61 and 4.76, Agreeableness between 5.11 and 5.29, Conscientiousness between 5.53 and 5.79, Emotional Stability between 5.39 and 5.63 and Openness to Experience between 5.13 and 5.37. The standard deviations of the five factors of the TIPI have values from 0.74 to 1.64. Similar to the TEIQue-SF questionnaire, in summary, the mean values of all factors present homogenous distribution within all the groups/subgroups, showing minimal deviations and, thus, stable results. 

### 3.3. Validation via Subjective Feedback

Following, the evaluations obtained from the subjective feedback of the participants are presented for Group A, Group B1, Group B2, and Group B3. They include the analysis of the SAM Ratings and the Direct Questions. The evaluation of the subjective feedback is necessary to provide ground truth and validation of the dataset, which is in turn essential for further analysis and applications. The Free Speech data are not analyzed here but are part of the dataset in their raw state.

#### 3.3.1. SAM Ratings

The SAM Ratings were collected from every subject after each accomplished sequence during the experiment. With the help of the three dimensions, Valence, Arousal, and Dominance, the induction of the different sequence levels of cognitive load and affective states is evaluated. First, the evaluation of the ratings of Group A is presented. Then, the ratings of the three different measurements of Group B are separately analyzed (Group B1, Group B2, and Group B3). Finally, repeated measures ANOVA and post-hoc corrections were performed to examine the significance of the variations between the different sequences.

**Figure 8:**
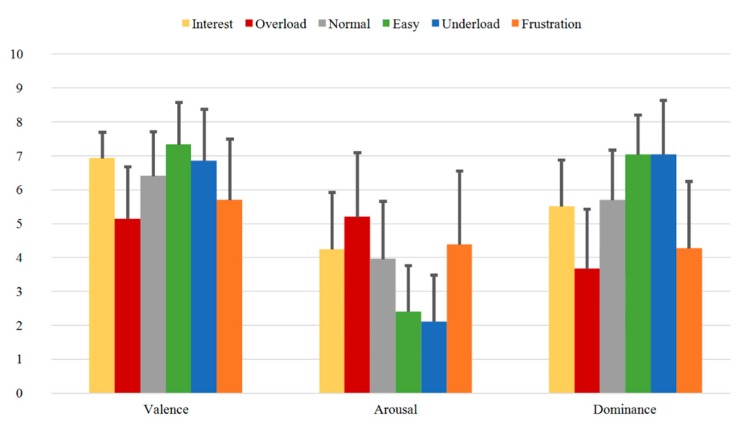
SAM Ratings of Group A for all sequences with mean Valence, Arousal, and Dominance.

In Figure 8, the mean SAM Ratings for Group A are presented. The highest valence values are found for the sequences *Easy* (7.32) and *Interest* (6.92) and *Underload* (6.84), while the lowest values are found for the *Overload* (5.13) and the *Frustration* (5.68) sequences. On the other hand, the highest Arousal was perceived for these two latter sequences, *Overload* (5.18) and *Frustration* (4.37), while the lowest Arousal was registered for *Underload* (2.11) and *Easy* (2.39). As for the Dominance values, the highest mean values were also obtained for these two sequences, *Underload* and *Easy* (7.03 each), while the lowest values were registered for *Overload* (3.66) and *Frustration* (4.26).

**Figure 9:**
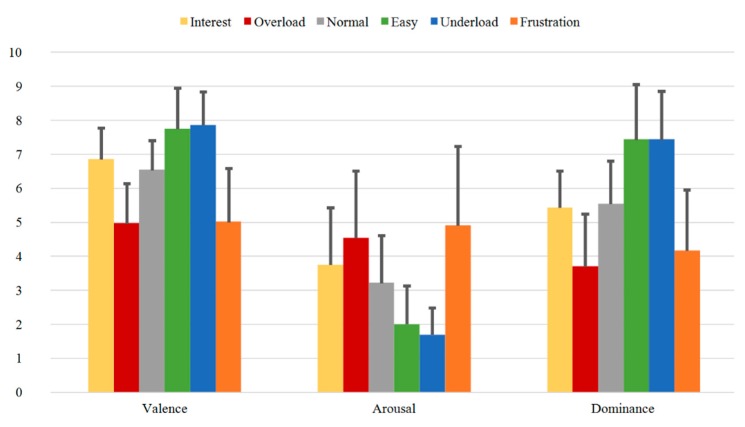
SAM Ratings of Group B1 for all sequences with mean Valence, Arousal, and Dominance.

**Figure 10:**
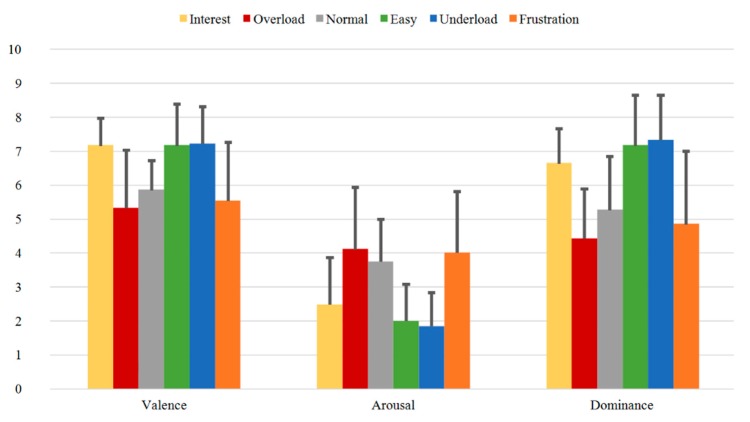
SAM Ratings of Group B2 for all sequences with mean Valence, Arousal, and Dominance.

**Figure 11:**
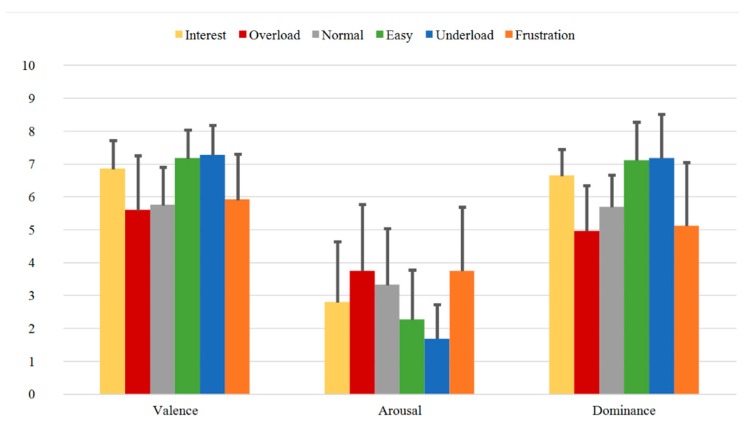
SAM Ratings of Group B3 for all sequences with mean Valence, Arousal, and Dominance.

Figure 9, Figure 10 and Figure 11 illustrate the SAM Ratings results for the first (Group B1), second (Group B2), and third (Group B3) measurement time of Group B, respectively. Additionally, here, the mean SAM Ratings values are consistent with each other for all three measurement times showing transtemporal stability in the subjective evaluation. Additionally, compared to the rating results of the one-measurement group (Group A) illustrated in Figure 8, the mean Valence, Arousal, and Dominance values show similar rating tendencies. 

In summary, the SAM Ratings show overall stable course with highest Valence, lowest Arousal, and highest Dominance values for the *Easy* and *Underload* sequences and with lowest Valence, highest Arousal and lowest Dominance values for the *Overload* and *Frustration* sequences. Both transtemporal stability and the similar rating tendencies between the subjects of the first and second samples prove the robust quality of the induction as evaluated by the subjects.

**Figure A1:**
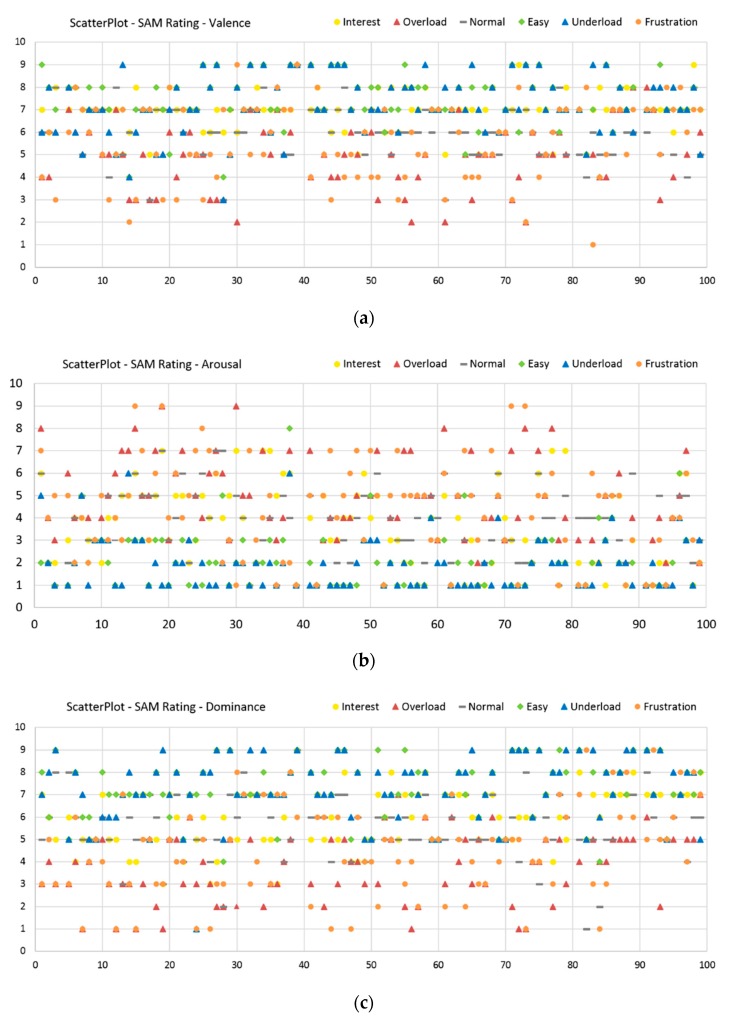
(**a**) Ratings distribution of the Valence dimension for all the measurements. (**b**) Ratings distribution of the Arousal dimension for all the measurements. (**c**) Ratings distribution of the Dominance dimension for all the measurements.

The distribution of the SAM Ratings for all the measurements are presented as scatter-plots in the Appendix A in Figure A1a (Valence), Figure A1b (Arousal), and Figure A1c (Dominance). 

In order to examine if the differences of the SAM Ratings evaluations are statistically significant in the VAD-space between the different sequences, further statistical analysis was carried out. To analyze the ratings for Group A and Group A + Group B1, we conducted separate repeated measures ANOVA with the factors Sequence and VAD (for Valence, Arousal, and Dominance, respectively). Post-hoc, Newman-Keuls corrections were carried out to compare the mean differences between the sequences. For Group A, the repeated measures ANOVA revealed a significant effect of Sequence (F(5.185) = 16.866, *p* < 0.001, ηp2 = 0.313), VAD (F(2.74) = 57.996, *p* < 0.001, ηp2 = 0.611) and the interaction (F(10.370 = 30.748, *p* < 0.001, ηp2 = 0.454). Additionally, post-hoc tests using Newman-Keuls correction revealed significant differences (see Table A1a in the Appendix A). For the combined Group A + Group B1, the repeated measures ANOVA revealed a significant effect of Sequence (F(5.280) = 30.190, *p* < 0.001, ηp2 = 0.353), VAD (F(2.112) = 106.429, *p* < 0.001, ηp2 = 0.774) and the interaction (F(10.560) = 51.405, *p* < 0.001, ηp2 = 0.447). Again, post-hoc tests using Newman-Keuls correction revealed significant differences (see entire Table A1b in the Appendix A). 

A direct analysis of the SAM Ratings of all sequences in comparison to the *Normal* sequence as baseline is presented in Table 3, while the results of the SAM Ratings between the *Overload* vs. *Underload* sequences and between the *Interest* vs. *Frustration* sequences are presented in Table 4 (the entire results can be found in the Appendix A as Table A1a and Table A1b).

Further, in order to justify the combination of Group A and Group B1 (all first measurements) in the statistical analysis, an ANOVA was additionally computed for the Valence, Arousal, and Dominance scores of the SAM Ratings between Group A and Group B1. Based on a one-way ANOVA, we found no statistically significant difference in the Valence scores (F(2.6) = 1.650, *p* = 0.153), nor in the Arousal scores (F(2.6) = 0.978, *p* = 0.450) nor in the Dominance scores (F(2.6) = 0.376, *p* = 0.891) between Group A and Group B1.

According to Table 3, most of the Valence, Arousal, and Dominance values of the SAM Ratings can be significantly distinguished from each other for all the sequences compared to *Normal*. Exceptions for Group A + Group B1 are the Valence, Arousal, and Dominance of *Interest* and the Valence of *Underload*. For Group A, more exceptions could be observed especially on the Valence dimension. More context-relevant results are the implications in Table 4, showing that the states *Overload* vs. *Underload* and *Interest* vs. *Frustration* can be significantly distinguished from each other on all SAM dimensions for both the Group A and the Group A + Group B1 except for Arousal between *Interest* and *Frustration*. 

#### 3.3.2. Direct Questions

A further subjective feedback evaluation was carried out in terms of Direct Questions. Therefore, after each sequence, the subjects were asked to answer Direct Questions related to the assessment of their own perception. Four questions related to “Difficulty”, “Performance”, “Stress”, and “Motivation” were processed: With the help of the first question, the subjects described how difficult the sequence was (very easy = 1; very difficult = 10). The second question is a personal performance assessment (performed very bad = 1; performed very well = 10). For the first sequence of *Interest*, this “Performance” question was adapted to answer the subjects’ interest. The third question describes the individually experienced stress level (very relaxed = 1; very stressed = 10), and the fourth question reflects the motivation of the participant (not motivated = 1; very motivated = 10).

**Figure 12:**
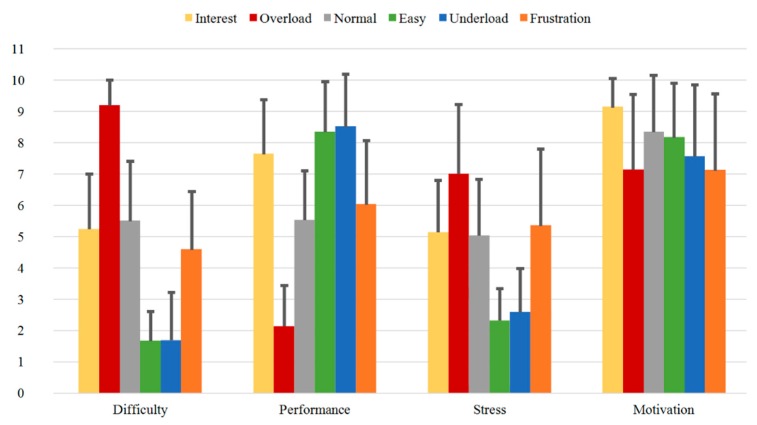
Direct Questions of Group A for all the sequences with mean values.

In Figure 12 the results of the Direct Questions are shown for Group A. It can be seen, that for the first question “Difficulty”, *Overload* has the highest rating (9.18), while *Easy* and *Underload* have the lowest ratings (1.66 and 1.68, respectively). As expected, the sequences *Interest*, *Normal*, and *Frustration* present middle ratings (5.24, 5.50, and 4.58, respectively). As for the second question “Performance”, the lowest rating is observed for *Overload* (2.13), while the highest ratings were obtained for *Easy* and *Underload* (8.34 and 8.50, respectively). The “Interest” rating for the first sequence *Interest* was 7.63. Further, the third “Stress” question shows similar course as the first “Difficulty” question. The “Stress” ratings for the sequences *Interest*, *Normal* and *Frustration* are in the same range (5.13, 5.03 and 5.34, respectively), while *Overload* has the highest rating (7.00) and *Easy* and *Underload* the lowest ones (2.32 and 2.58, respectively). An interesting observation here, is the slightly increasing stress from *Easy* to *Underload*. The last “Motivation” question shows the highest rating for *Interest* (9.13), and the lowest ratings for *Overload* (7.13), *Underload* (7.55), and *Frustration* (7.11). 

**Figure 13:**
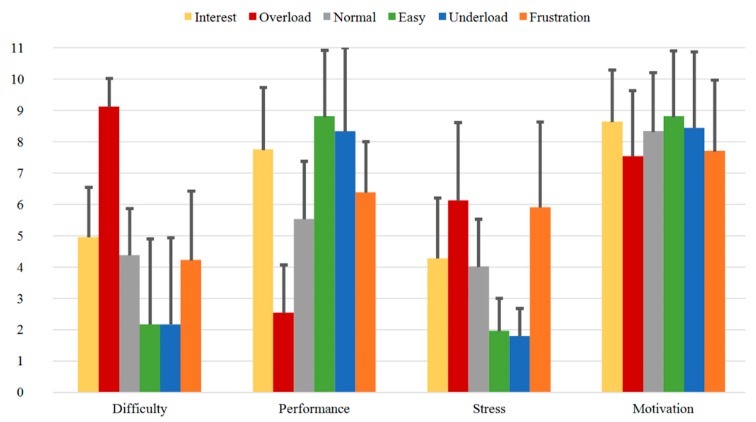
Direct Questions of Group B1 for all the sequences with mean values.

**Figure 14:**
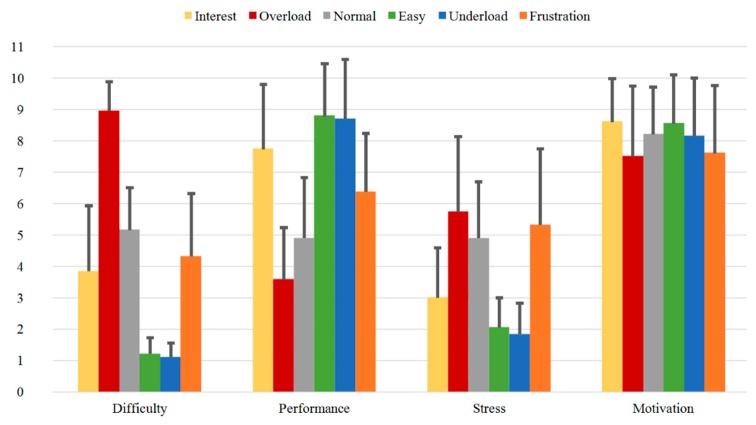
Direct Questions of Group B2 for all the sequences with mean values.

**Figure 15:**
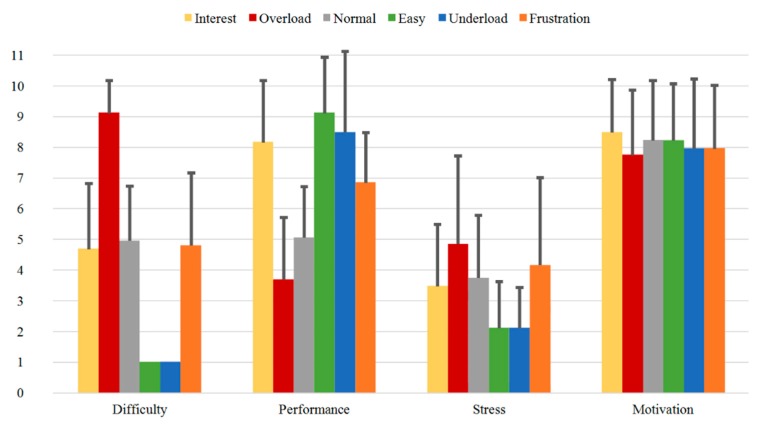
Direct Questions of Group B3 for all the sequences with mean values.

With regard to Group B with the subjects who underwent three measurements each, some changes over time can be observed. Figure 13, Figure 14 and Figure 15 illustrate the Direct Questions ratings for the first (Group B1), second (Group B2), and third (Group B3) measurement, respectively. The mean rating distributions for each sequence for Group B1 (first measurement) presented in Figure 13 are comparable to the results obtained for Group A (single measurement) presented in Figure 12.

Comparing Group B1 and Group B2, the mean rating values of the first question “Difficulty” for the sequences *Interest*, *Easy* and *Underload* decrease from the first to the second measurement. On the other hand, the mean rating values for *Normal* increase from 4.37 to 5.16. As for the second “Performance” question, the mean rating values for *Overload* (2.53 vs. 3.58) and *Underload* (8.32 vs. 8.68) increase from the first to the second measurement, while the rating related to *Normal* decreases (5.53 vs. 4.89). As for the third “Stress” question, higher differences are observed for the *Interest* (4.26 vs. 3.00), *Normal* (4.00 vs. 4.89) and *Frustration* (5.89 vs. 5.32) sequences. Finally, the last “Motivation” question has comparable tendencies and values for the first and second measurements.

Finally, comparing Group B2 and Group B3 illustrated in Figure 14 and Figure 15, the mean values of the “Difficulty” question for the sequences *Interest*, *Overload* and *Frustration* increase (3.84 vs. 4.68, 8.95 vs. 9.11 and 4.32 vs. 4.79, respectively), while the values for *Normal*, *Easy* and *Underload* decrease (5.16 vs. 4.95, 1.21 vs. 1.00 and 1.11 vs. 1.00, respectively). The highest rating in the third measurement is again obtained for the sequence *Overload* (9.11), while the lowest values are observed for the sequences *Easy* and *Underload* with values of 1.00 each. Furthermore, the “Performance” question shows increasing values for *Interest* from the second to the third measurement time (7.74 vs. 8.16), while the related mean ratings of the remaining sequences have nearly the same values with small variations. As for the “Stress” question, the highest mean values are also observed for the *Overload* and *Frustration* sequences and show a decreasing tendency compared to the second measurement (5.74 vs. 4.84 and 5.32 vs. 4.16, respectively). The “Motivation” question for all sequences results in mean ratings ranging around the value of 8, with the lowest values obtained for the sequences *Overload* (7.50 vs. 7.74) and *Frustration* (7.60 vs. 7.95). 

**Figure A2:**
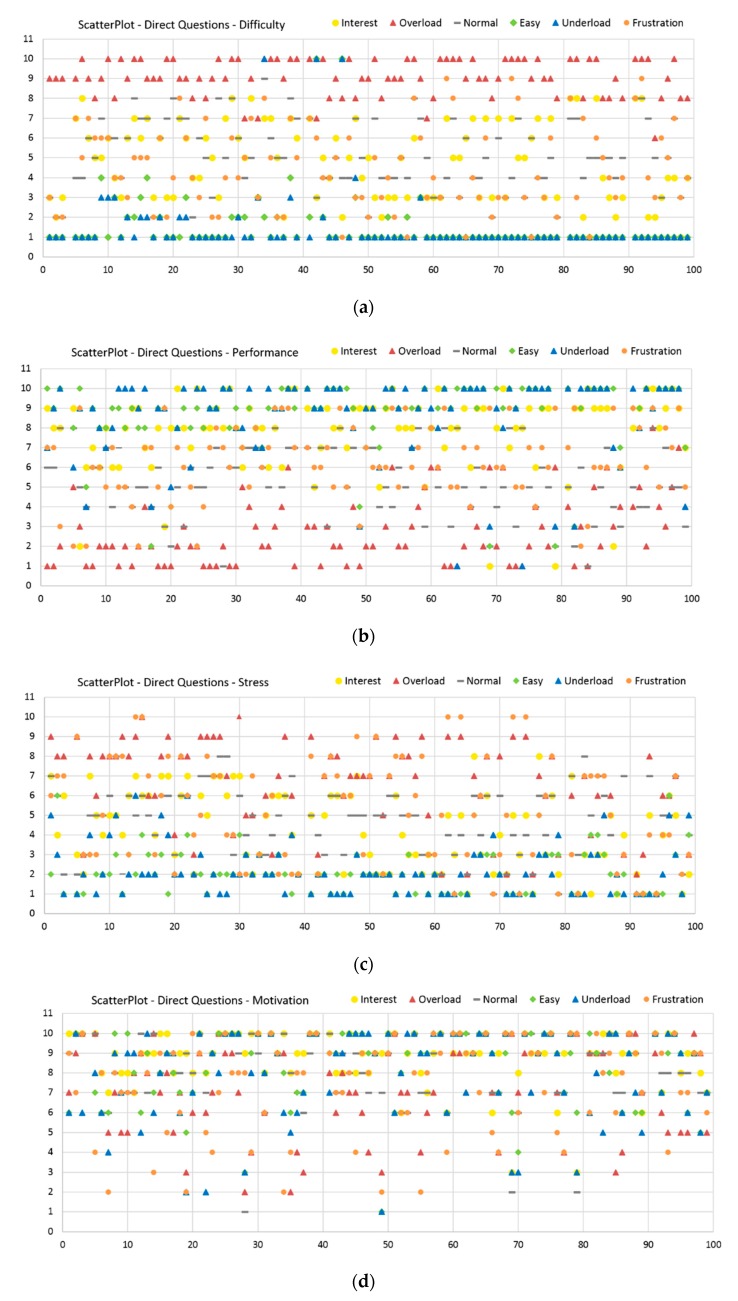
(**a**) Ratings distribution of the “Difficulty” question for all the measurements. (**b**) Ratings distribution of the “Performance” question for all the measurements. (**c**) Ratings distribution of the “Stress” question for all the measurements. (**d**) Ratings distribution of the “Motivation” question for all the measurements.

The ratings distribution of the Direct Questions for all the measurements are presented as scatter-plots in the Appendix A in Figure A2a (“Difficulty”), Figure A2b (“Performance”), Figure A2c (“Stress”), and Figure A2d (“Motivation”). 

In order to examine if the differences of the Direct Questions evaluations are statistically significant between the different sequences, further statistical analysis was carried out. Similar to the SAM Ratings, we conducted separate repeated measures ANOVA with a post-hoc Newman-Keuls correction to analyze differences between the respective ratings of the individual questions “Difficulty” (Dif), “Performance” (Per), “Stress” (Str) and “Motivation” (Mot) for Group A and Group A + Group B1. For Group A, the repeated measures ANOVA revealed a significant effect of Sequence (F(5.185) = 43.379, *p* < 0.001, ηp2 = 0.540), Question (F(3.111) = 74.360, *p* < 0.001, ηp2 = 0.668) and the interaction (F(15.555 = 81.485, *p* < 0.001, ηp2 = 0.688). Additionally, post-hoc tests using Newman-Keuls correction revealed significant differences (see Table A2a in the Appendix A). For the combined Group A + Group B1, the repeated measures ANOVA revealed a significant effect of Sequence (F(5.280) = 35.164, *p* < 0.001, ηp2 = 0.386), Question (F(3.168) = 126.204, *p* < 0.001, ηp2 = 0.693) and the interaction (F(15.840) = 111.873, *p* < 0.001, ηp2 = 0.666). Again, post-hoc tests using Newman-Keuls correction revealed significant differences (see Table A2b in the Appendix A). 

A direct analysis of the Direct Questions of all sequences in comparison to the *Normal* sequence as baseline is presented in Table 5, while the results of the Direct Questions between the *Overload* vs. *Underload* sequences and between the *Interest* vs. *Frustration* sequences are presented in Table 6 (the entire results can be found in the Appendix A as Table A2a and Table A2b).

Further, in order to justify the combination of Group A and Group B1 (all first measurements) in the statistical analysis, an ANOVA was additionally computed for the individual ratings of the Direct Questions between Group A and Group B1. Based on a one-way ANOVA, we did not find any statistically significant difference in the “Difficulty” scores (F(2.6) = 1.333, *p* = 0.260), nor in the “Performance” (F(2.6) = 0.6778, *p* = 0.668), nor in the “Stress” (F(2.6) = 1.740, *p* = 0.131), nor in the “Motivation” (F(2.6) = 1.072, *p* = 0.392) scores between Group A and Group B1.

According to Table 5, most of the Direct Questions can be significantly distinguished from each other for all the sequences compared to *Normal*. Exceptions are the “Difficulty” and “Stress” questions of *Interest* and *Frustration* as well as the “Motivation” question of *Interest, Easy* and *Underload* for both Group A and Group A + Group B1, in addition to the “Performance” question of *Frustration* for Group A. More context-relevant results are the implications in Table 6, which show that the states *Overload* vs. *Underload* and *Interest* vs. *Frustration* can be significantly distinguished from each other for all the Direct Questions except for the “Difficulty” and “Stress” question between *Interest* vs. *Frustration* and the “Motivation” question between *Overload* vs. *Underload* for both Group A and Group A + Group B1.

### 3.4. Data Annotation

In addition to the basic annotation, leading from the experimental design and application log files, the dataset is enhanced by various semi-automatic generated labels. The basic annotation contains the exact timing information at millisecond level of the beginning and ending of all sequences: Timestamps when each search item was presented, if and when a subject pronounced the solution, including whether the solution was correct or wrong and all information of the given subjective feedback. These mostly technical annotations do not necessarily contain emotional information, although they can give hints on situations where the probability of emotional reactions raises, for instance in case of timeouts or wrong answers, or during maximum load phases.

The semi-automatic labels are generated by our data driven active learning approach, presented in [62,63]. The basic assumption of this approach is the sparseness of emotional reactions in the audio and video modalities. In several pre-studies we figured out, that in HCI scenarios, users mostly tend to have emotions only in a few situations, or at least show them only in sparseness [64]. This leads to the assumption that most of the recorded data represent neutral emotional content. Based on this assumption, we train different density estimation models, such as One Class SVM, SVDD, or GMM on the whole dataset (ignoring the underling experimental structure) and then compare each feature vector instance with this neutral or background model. If a specific feature vector has a high distance compared to the background model, the probability of having an emotional instance increases. As such, less fitting-points are then presented to experts, which rate the points towards the emotional content. After having the first points labeled (emotion or neutral), these labels are used to improve the background model iteratively, until most of the outlier data points are labeled. Details of this active learning-based process can be found in the cited papers. The main conclusion of our active learning algorithms is the dramatic reduction of annotation effort in case of affective datasets like the one presented here. In most cases, only 10% of a naturalistic HCI dataset has to be annotated in order to achieve the same classification results as the baseline classifier using the full dataset. The active learning based, semi-automatic generated labels are part of the dataset. Further we had to manually label nine participants in order to evaluate our active learning approach. These manual labels are also part of the dataset.

Additionally, we provide some further manual created labels regarding the body pose information. As described in [65], we annotated several body poses based on distance measures of the skeleton provided by the Kinect sensor. Static poses include onsets and offsets of: arms crossed, hands behind back, hands on hips, legs crossed, and legs in step position. Dynamic poses include: sideways moving hands away from body, facial hand touch, and quick movement of feet.

## 4. Discussion and Summary

The resulting multimodal *uulmMAC* database from our emotional and cognitive load scenario conducted in a mobile interactive HCI setting is a valuable contribution to research fields related to multimodal affective computing and machine learning applications in HCI. Summarized, the main contributions of our work include the following:

- **Dataset for affective computing research:** We designed and implemented a HCI scenario and acquired the *uulmMAC* dataset for emotional and cognitive states recognition. The dataset consists of six different sequences, including the states *Interest, Overload, Normal, Easy, Underload*, and *Frustration*. The emotional-cognitive conditions were thereby induced by increasing the task field objects and colors as well as decreasing the available time of an interactive game paradigm.
- **Multimodal mobile and interactive:** It consists of highly multimodal (biosignals, videos, audios, Kinect), mobile (standing, walking, freely moving positions with wireless physiology sensors), and interactive (HCI via natural speech) emotional-cognitive HCI scenario.
- **Large number of subjects/recording sessions:** The original experiment includes 60 subjects and 100 recording sessions, from which 57 subjects and 95 recording sessions are left as part of the final dataset. Depending on the focus of the research question or modality, the recording sessions of 38 subjects or 19 subjects or all 57 subjects can be analyzed: For instance, when focusing on physiological reactions with no specific interest in facial EMG, all 57 subjects (Group A + Group B1) can be analyzed; while when focusing on facial expression from video data, 38 subjects (Group A) can be analyzed.
- **Transtemporal analysis:** Our dataset also allows transtemporal research and investigations of the changes and variations of the induction, reactions and recognition over time: This is possible with our second sample of 20 subjects who underwent three different measurements at three different times with one-week interval-time inbetween. The transtemporal part of the final dataset includes 19 subjects (Group B) out of the original 20 subjects, left after quality check. Further, the data analysis and evaluation of the subjective feedback and questionnaires show transtemporally stable and valid induction results.
- **Validated dataset:** The induction of the various emotional and cognitive load states via six different sequences is validated through evaluation of subjective feedback acquired during the experiment. These are used as ground truth for our paradigm. On one side, the reported SAM Ratings vary between the sequences and show significant differences between the relevant induced states (Table 3 and Table 4 or Table A1a and Table A1b). Additionally, the SAM Ratings results of Group B1 show a consistent course with the results of Group A (first measurements from both samples) with no statistically significant differences based on an ANOVA. On the other side, the results from the Direct Questions are compatible with the related induction state (i.e., the *Overload* and *Frustration* sequences have high “Stress” answers rates, while the *Interest* sequence has high “Motivation” answers rates etc.) and show significant differences between the relevant induced states (Table 5 and Table 6 or Table A2a and Table A2b). Additionally, the Direct Questions ratings of Group B1 present similar course as the results of Group A for all the four questions with no statistically significant differences based on an ANOVA. Finally, the evaluation and analysis of the various questionnaires, acquired from the subjects prior the experiment, also show stable results.
- **High technical quality:** The technical quality of the data and related signals is also checked and demonstrated via different preliminary classifications conducted on various subsets of the database including: the video data [63], the gesture data [65], the audio data [66], the biophysiological data [67], the speech and the biophysiological data [68], and the multimodal data [69].

Overall, we created a dataset for various applications in the fields of affective computing and machine learning, including classifications, feature analysis, multimodal fusion or transtemporal investigations. The dataset includes multimodal sensor data as well as various annotations and extracted labels. Limitations of this work include the relatively limited number of transtemporal data (57 measurements from 19 subjects) as well as the absence of electroencephalography (EEG) or electrooculography (EOG) data for brain and eye movement analysis, both relevant for cognitive reactions. Finally, the experiment was conducted in a laboratory setting designed to be close to real HCI, and the next step would be to transfer our settings and findings into-the-wild for closer real-life induction and recognition research. 

Future work will include numerical evaluations based on classification models using machine learning for the full dataset. Thereby, standardized sets of feature extraction techniques for each recorded modality will be generated and standard features for each emotional and cognitive state will be defined. A multimodal fusion analysis will be conducted to investigate the effect of each modality on the recognition rates of the different states. Further, a transtemporal analysis of the Group B data will be conducted to investigate the changes in time including features and classifications. Further, investigations related to the analysis of human-computer dialogs could be conducted, for instance to investigate the effects of computer feedbacks on human performance and the psychophysiological responses. Similarly, a gender analysis could also be conducted to investigate differences in the elicitation levels, emotional-cognitive psychophysiological responses or in the recognition rates and individual performance. 

Finally, considering the relevance of emotional *Frustration* and cognitive *Overload* in the emergence of stress, which was investigated in many studies [70,71,72,73], we believe that our *uulmMAC* database on emotional and cognitive load states can also be used for affective computing and machine learning applications in the field of stress research. The well-adapted TSST—Trier Social Stress Test [70] employs a mental arithmetic task to induce high cognitive load (beside a social-evaluative part based on a public speaking task). The Stroop Color Test [71] employs a word-color task to induce high cognitive load and was further adopted by Choi et al. [74] in their experiments to develop a wearable stress monitoring system. Additionally, Wijsman et al. employ computer tasks (calculation, puzzle, memorization) under time pressure to induce stress [72]. In a similar context, a multimodal dataset was recently collected within the SWELL project [73] to induce stress by manipulating the working conditions of the subjects through mail interruptions and time pressure. Based on these studies, we will investigate in our future work the application of our database to the field of stress recognition research. It would be of interest if specialized machine learning techniques like transfer learning and/or deep learning approaches can be applied to transfer features and classifiers created on the *uulmMAC* dataset into the stress classification scenario.

## Figures and Tables

**Table 1:** Illustration of the experimental procedure and sequences description.

Sequence Nr.	Induction of	Number of Tasks	Search Field	Time (s) to Answer
*Sequence 1	Interest	40 tasks	3 × 3 and 4 × 4 items	10 s/task
*Sequence 2	Overload	40 tasks	6 × 6 items	6 s/task
*Sequence 3	Normal	40 tasks	4 × 4 items	10 s/task
*Sequence 4	Easy	40 tasks	3 × 3 items	100 s/task
*Sequence 5	Underload	40 tasks	3 × 3 items	100 s/task
*Sequence 6	Frustration	40 tasks	3 × 3 and 4 × 4 items	10 s/task

*(Every) Sequence is followed by subjective feedback, respiration baseline, and results’ summary.

**Table 2:** Average network latency between recording modules and estimated maximum delay between modalities.

Modality	Average Network Latency (ms)	Estimated Max. Delay (ms)
Kinect (Video / Depth / IR / Audio / Pose)	5.61	1.35
Front video	4.95	1.27
Rear video, atmosphere audio, headset audio, biosignals	5.37	0.83
User interface	5.17	1.17
WOZ interface	5.52	1.17
**Average estimated synchronization error**		1.35 – 0.83 = **0.52**

**Table 3:** Post-hoc Newman-Keuls corrections for the Valence (V), Arousal (A), and Dominance (D) ratings between all sequences compared to *Normal*. Mean-Differences (Mean-Diff.) and *p*-values are presented.

SAM Ratings	Mean-Diff. Group A	*p*-Value Group A	Mean-Diff. Group A + Group B1	*p*-Value Group A + Group B1
V_Normal – V_Interest	–0.526	0.272	–0.509	0.100
V_Normal – V_Overload	1.263	0.003**	1.351	0.000***
V_Normal – V_Easy	–0.921	0.076	–1.053	0.003**
V_Normal – V_Underload	–0.447	0.190	–0.772	0.061
V_Normal – V_Frustration	0.711	0.094	0.930	0.002**
A_Normal – A_Interest	–0.289	0.397	–0.386	0.184
A_Normal – A_Overload	–1.237	0.004**	–1.246	0.000***
A_Normal – A_Easy	1.553	0.000***	1.491	0.000***
A_Normal – A_Underload	1.842	0.000***	1.754	0.000***
A_Normal – A_Frustration	–0.421	0.606	–0.807	0.013*
D_Normal – D_Interest	0.184	0.852	0.175	0.569
D_Normal – D_Overload	2.026	0.000***	2.018	0.000***
D_Normal – D_Easy	–1.342	0.001**	–1.561	0.000***
D_Normal – D_Underload	–1.342	0.001**	-1.579	0.000***
D_Normal – D_Frustration	1.421	0.001**	1.404	0.000***

*p < 0.05, **p < 0.01, ***p < 0.001.

**Table 4:** Post-hoc Newman-Keuls corrections for the Valence (V), Arousal (A), and Dominance (D) ratings between *Overload* vs. *Underload* and between *Interest* vs. *Frustration*. Mean-Differences (Mean-Diff.) and *p*-values are presented.

SAM Ratings	Mean-Diff. Group A	*p*-Value Group A	Mean-Diff. Group A + Group B1	*p*-Value Group A + Group B1
V_Overload – V_Underload	–1.711	0.000***	–2.123	0.000***
V_Interest – V_Frustration	1.237	0.003**	1.439	0.000***
A_Overload – A_Underload	3.079	0.000***	3.000	0.000***
A_Interest – A_Frustration	–0.132	0.921	–0.421	0.202
D_Overload – D_Underload	–3.368	0.000***	–3.596	0.000***
D_Interest – D_Frustration	1.237	0.003**	1.228	0.000***

*p < 0.05, **p < 0.01, ***p < 0.001.

**Table 5:** Post-hoc Newman-Keuls corrections for the “Difficulty” (Dif), “Performance” (Per), “Stress” (Str) and “Motivation” (Mot) questions between all sequences compared to *Normal*. Mean-Differences (Mean-Diff.) and *p*-values are presented.

Direct Questions	Mean-Diff. Group A	*p*-Value Group A	Mean-Diff. Group A + Group B1	*p*-Value Group A + Group B1
Dif_Normal – Dif_Interest	0.263	0.742	–0.035	0.907
Dif_Normal – Dif_Overload	–3.684	0.000***	–3.965	0.000***
Dif_Normal – Dif_Easy	3.842	0.000***	3.351	0.000***
Dif_Normal – Dif_Underload	3.816	0.000***	3.333	0.000***
Dif_Normal – Dif_Frustration	0.921	0.103	0.719	0.080
Per_Normal – Per_Interest	–2.105	0.000***	–2.123	0.000***
Per_Normal – Per_Overload	3.395	0.000***	3.316	0.000***
Per_Normal – Per_Easy	–2.816	0.000***	–3.000	0.000***
Per_Normal – Per_Underload	–2.974	0.000***	–2.947	0.000***
Per_Normal – Per_Frustration	–0.500	0.162	–0.632	0.036*
Str_Normal – Str_Interest	–0.105	0.768	–0.123	0.684
Str_Normal – Str_Overload	–1.974	0.000***	–2.053	0.000***
Str_Normal – Str_Easy	2.711	0.000***	2.544	0.000***
Str_Normal – Str_Underload	2.447	0.000***	2.421	0.000***
Str_Normal – Str_Frustration	–0.316	0.813	–0.737	0.104
Mot_Normal – Mot_Interest	–0.789	0.070	–0.667	0.176
Mot_Normal – Mot_Overload	1.211	0.009**	1.035	0.005**
Mot_Normal – Mot_Easy	0.184	0.864	0.000	1.000
Mot_Normal – Mot_Underload	0.789	0.176	0.544	0.072
Mot_Normal – Mot_Frustration	1.237	0.010*	1.035	0.003**

*p < 0.05, **p < 0.01, ***p < 0.001.

**Table 6:** Post-hoc Newman-Keuls corrections for the “Difficulty” (Dif), “Performance” (Per), “Stress” (Str) and “Motivation” (Mot) questions between *Overload* vs. *Underload* and *Interest* vs. *Frustration*. Mean-Differences (Mean-Diff.) and *p-*values are presented.

Direct Questions	Mean-Diff. Group A	*p*-Value Group A	Mean-Diff. Group A + Group B1	*p*-Value Group A + Group B1
Dif_Overload – Dif_Underload	7.500	0.000***	7.298	0.000***
Dif_Interest – Dif_Frustration	0.658	0.254	0.754	0.091
Per_Overload – Per_Underload	–6.368	0.000***	–6.263	0.000***
Per_Interest – Per_Frustration	1.605	0.000***	1.491	0.000***
Str_Overload – Str_Underload	4.421	0.000***	4.474	0.000***
Str_Interest – Str_Frustration	–0.211	0.826	–0.614	0.175
Mot_Overload – Mot_Underload	–0.421	0.239	–0.491	0.363
Mot_ Interest – Mot_Frustration	2.026	0.000***	1.702	0.000***

*p < 0.05, **p < 0.01, ***p < 0.001.

## Appendix A

Table A1 (**a**) Newman-Keuls post-hoc corrections for the VAD dimensions (V = Valence; A = Arousal; D = Dominance) of the SAM Ratings and the six sequences Seq. (I= *Interest*, O= *Overload*, N= *Normal*, E= *Easy*, U= *Underload*, F= *Frus**tration*) for Group A. Significant effects are highlighted in bold (*p* < 0.05). (**b**) Newman-Keuls post-hoc corrections for the VAD dimensions (V = Valence; A = Arousal; D = Dominance) of the SAM Ratings and the six sequences Seq. (I= *Interest*, O= *Overload*, N= *Normal*, E= *Easy*, U= *Underload*, F= *Frus**tration*) for Group A + Group B1. Significant effects are highlighted in bold (*p* < 0.05).

**Table:** (**a**)

**VAD**		**V**	**V**	**V**	**V**	**V**	**V**	**A**	**A**	**A**	**A**	**A**	**A**	**D**	**D**	**D**	**D**	**D**	**D**
	**Seq.**	**I**	**O**	**N**	**E**	**U**	**F**	**I**	**O**	**N**	**E**	**U**	**F**	**I**	**O**	**N**	**E**	**U**	**F**
**V**	**I**		**0.000**	0.272	0.655	0.817	**0.003**	**0.000**	**0.000**	**0.000**	**0.000**	**0.000**	**0.000**	**0.000**	**0.000**	**0.002**	0.758	0.949	**0.000**
**V**	**O**	**0.000**		**0.003**	**0.000**	**0.000**	0.369	**0.044**	0.878	**0.005**	**0.000**	**0.000**	**0.025**	0.527	**0.000**	0.486	**0.000**	**0.000**	**0.030**
**V**	**N**	0.272	**0.003**		0.076	0.190	0.094	**0.000**	**0.004**	**0.000**	**0.000**	**0.000**	**0.000**	**0.044**	**0.000**	**0.038**	0.250	0.345	**0.000**
**V**	**E**	0.655	**0.000**	0.076		0.636	**0.000**	**0.000**	**0.000**	**0.000**	**0.000**	**0.000**	**0.000**	**0.000**	**0.000**	**0.000**	0.673	0.397	**0.000**
**V**	**U**	0.817	**0.000**	0.190	0.636		**0.004**	**0.000**	**0.000**	**0.000**	**0.000**	**0.000**	**0.000**	**0.001**	**0.000**	**0.002**	0.852	0.949	**0.000**
**V**	**F**	**0.003**	0.369	0.094	**0.000**	**0.004**		**0.000**	0.309	**0.000**	**0.000**	**0.000**	**0.001**	0.590	**0.000**	1.000	**0.001**	**0.002**	**0.000**
**A**	**I**	**0.000**	**0.044**	**0.000**	**0.000**	**0.000**	**0.000**		**0.044**	0.397	**0.000**	**0.000**	0.921	**0.003**	0.207	**0.001**	**0.000**	**0.000**	0.939
**A**	**O**	**0.000**	0.878	**0.004**	**0.000**	**0.000**	0.309	**0.044**		**0.004**	**0.000**	**0.000**	**0.045**	0.355	**0.000**	0.460	**0.000**	**0.000**	**0.035**
**A**	**N**	**0.000**	**0.005**	**0.000**	**0.000**	**0.000**	**0.000**	0.397	**0.004**		**0.000**	**0.000**	0.606	**0.000**	0.397	**0.000**	**0.000**	**0.000**	0.625
**A**	**E**	**0.000**	**0.000**	**0.000**	**0.000**	**0.000**	**0.000**	**0.000**	**0.000**	**0.000**		0.397	**0.000**	**0.000**	**0.000**	**0.000**	**0.000**	**0.000**	**0.000**
**A**	**U**	**0.000**	**0.000**	**0.000**	**0.000**	**0.000**	**0.000**	**0.000**	**0.000**	**0.000**	0.397		**0.000**	**0.000**	**0.000**	**0.000**	**0.000**	**0.000**	**0.000**
**A**	**F**	**0.000**	**0.025**	**0.000**	**0.000**	**0.000**	**0.001**	0.921	**0.045**	0.606	**0.000**	**0.000**		**0.005**	0.229	**0.002**	**0.000**	**0.000**	0.758
**D**	**I**	**0.000**	0.527	**0.044**	**0.000**	**0.001**	0.590	**0.003**	0.355	**0.000**	**0.000**	**0.000**	**0.005**		**0.000**	0.852	**0.000**	**0.000**	**0.003**
**D**	**O**	**0.000**	**0.000**	**0.000**	**0.000**	**0.000**	**0.000**	0.207	**0.000**	0.397	**0.000**	**0.000**	0.229	**0.000**		**0.000**	**0.000**	**0.000**	0.287
**D**	**N**	**0.002**	0.486	**0.038**	**0.000**	**0.002**	1.000	**0.001**	0.460	**0.000**	**0.000**	**0.000**	**0.002**	0.852	**0.000**		**0.001**	**0.001**	**0.001**
**D**	**E**	0.758	**0.000**	0.250	0.673	0.852	**0.001**	**0.000**	**0.000**	**0.000**	**0.000**	**0.000**	**0.000**	**0.000**	**0.000**	**0.001**		1.000	**0.000**
**D**	**U**	0.949	**0.000**	0.345	0.397	0.949	**0.002**	**0.000**	**0.000**	**0.000**	**0.000**	**0.000**	**0.000**	**0.000**	**0.000**	**0.001**	1.000		**0.000**
**D**	**F**	**0.000**	**0.030**	**0.000**	**0.000**	**0.000**	**0.000**	0.939	**0.035**	0.625	**0.000**	**0.000**	0.758	**0.003**	0.287	**0.001**	**0.000**	**0.000**

**Table:** (**b**)

**VAD**		**V**	**V**	**V**	**V**	**V**	**V**	**A**	**A**	**A**	**A**	**A**	**A**	**D**	**D**	**D**	**D**	**D**	**D**
	**Seq.**	**I**	**O**	**N**	**E**	**U**	**F**	**I**	**O**	**N**	**E**	**U**	**F**	**I**	**O**	**N**	**E**	**U**	**F**
**V**	**I**		**0.000**	0.100	0.254	0.742	**0.000**	**0.000**	**0.000**	**0.000**	**0.000**	**0.000**	**0.000**	**0.000**	**0.000**	**0.000**	0.343	0.609	**0.000**
**V**	**O**	**0.000**		**0.000**	**0.000**	**0.000**	0.164	**0.003**	0.704	**0.000**	**0.000**	**0.000**	0.139	0.313	**0.000**	0.179	**0.000**	**0.000**	**0.013**
**V**	**N**	0.100	**0.000**		**0.003**	0.061	**0.002**	**0.000**	**0.000**	**0.000**	**0.000**	**0.000**	**0.000**	**0.001**	**0.000**	**0.004**	**0.026**	**0.047**	**0.000**
**V**	**E**	0.254	**0.000**	**0.003**		0.311	**0.000**	**0.000**	**0.000**	**0.000**	**0.000**	**0.000**	**0.000**	**0.000**	**0.000**	**0.000**	0.705	0.529	**0.000**
**V**	**U**	0.742	**0.000**	0.061	0.311		**0.000**	**0.000**	**0.000**	**0.000**	**0.000**	**0.000**	**0.000**	**0.000**	**0.000**	**0.000**	0.998	0.950	**0.000**
**V**	**F**	**0.000**	0.164	**0.002**	**0.000**	**0.000**		**0.000**	0.179	**0.000**	**0.000**	**0.000**	**0.006**	0.950	**0.000**	0.802	**0.000**	**0.000**	**0.000**
**A**	**I**	**0.000**	**0.003**	**0.000**	**0.000**	**0.000**	**0.000**		**0.007**	0.184	**0.000**	**0.000**	0.202	**0.000**	0.313	**0.000**	**0.000**	**0.000**	0.569
**A**	**O**	**0.000**	0.704	**0.000**	**0.000**	**0.000**	0.179	**0.007**		**0.000**	**0.000**	**0.000**	0.129	0.257	**0.000**	0.114	**0.000**	**0.000**	**0.022**
**A**	**N**	**0.000**	**0.000**	**0.000**	**0.000**	**0.000**	**0.000**	0.184	**0.000**		**0.000**	**0.000**	**0.013**	**0.000**	0.899	**0.000**	**0.000**	**0.000**	0.139
**A**	**E**	**0.000**	**0.000**	**0.000**	**0.000**	**0.000**	**0.000**	**0.000**	**0.000**	**0.000**		0.282	**0.000**	**0.000**	**0.000**	**0.000**	**0.000**	**0.000**	**0.000**
**A**	**U**	**0.000**	**0.000**	**0.000**	**0.000**	**0.000**	**0.000**	**0.000**	**0.000**	**0.000**	0.282		**0.000**	**0.000**	**0.000**	**0.000**	**0.000**	**0.000**	**0.000**
**A**	**F**	**0.000**	0.139	**0.000**	**0.000**	**0.000**	**0.006**	0.202	0.129	**0.013**	**0.000**	**0.000**		**0.007**	**0.014**	**0.001**	**0.000**	**0.000**	0.255
**D**	**I**	**0.000**	0.313	**0.001**	**0.000**	**0.000**	0.950	**0.000**	0.257	**0.000**	**0.000**	**0.000**	**0.007**		**0.000**	0.569	**0.000**	**0.000**	**0.000**
**D**	**O**	**0.000**	**0.000**	**0.000**	**0.000**	**0.000**	**0.000**	0.313	**0.000**	0.899	**0.000**	**0.000**	**0.014**	**0.000**		**0.000**	**0.000**	**0.000**	0.179
**D**	**N**	**0.000**	0.179	**0.004**	**0.000**	**0.000**	0.802	**0.000**	0.114	**0.000**	**0.000**	**0.000**	**0.001**	0.569	**0.000**		**0.000**	**0.000**	**0.000**
**D**	**E**	0.343	**0.000**	**0.026**	0.705	0.998	**0.000**	**0.000**	**0.000**	**0.000**	**0.000**	**0.000**	**0.000**	**0.000**	**0.000**	**0.000**		1.000	**0.000**
**D**	**U**	0.609	**0.000**	**0.047**	0.529	0.950	**0.000**	**0.000**	**0.000**	**0.000**	**0.000**	**0.000**	**0.000**	**0.000**	**0.000**	**0.000**	1.000		**0.000**
**D**	**F**	**0.000**	**0.013**	**0.000**	**0.000**	**0.000**	**0.000**	0.569	**0.022**	0.139	**0.000**	**0.000**	0.255	**0.000**	0.179	**0.000**	**0.000**	**0.000**	

Table A2 (**a**) Newman-Keuls post-hoc corrections for the Direct Questions Quest. (Dif = “Difficulty”; Per = “Performance”; Str = “Stress”; Mot = “Motivation”) and the sequences Seq. (I= *Interest*, O= *Overload*, N= *Normal*, E= *Easy*, U= *Underload*, F= *Frus**tration*) for Group A. Significant effects are marked bold (*p* < 0.05). (**b**) Newman-Keuls post-hoc corrections for the Direct Questions Quest. (Dif = “Difficulty”; Per = “Performance”; Str = “Stress”; Mot = “Motivation”) and the sequences Seq. (I= *Interest*, O= *Overload*, N= *Normal*, E= *Easy*, U= *Underload*, F= *Frus**tration*) for Group A + Group B1. Significant effects are marked bold (*p* < 0.05).

**Table:** (**a**)

**Quest.**		**Dif**	**Dif**	**Dif**	**Dif**	**Dif**	**Dif**	**Per**	**Per**	**Per**	**Per**	**Per**	**Per**	**Str**	**Str**	**Str**	**Str**	**Str**	**Str**	**Mot**	**Mot**	**Mot**	**Mot**	**Mot**	**Mot**
	**Seq.**	**I**	**O**	**N**	**E**	**U**	**F**	**I**	**O**	**N**	**E**	**U**	**F**	**I**	**O**	**N**	**E**	**U**	**F**	**I**	**O**	**N**	**E**	**U**	**F**
**Dif**	**I**		**0.000**	0.742	**0.000**	**0.000**	0.254	**0.000**	**0.000**	0.850	**0.000**	**0.000**	0.176	0.768	**0.000**	0.826	**0.000**	**0.000**	0.768	**0.000**	**0.000**	**0.000**	**0.000**	**0.000**	**0.000**
**Dif**	**O**	**0.000**		**0.000**	**0.000**	**0.000**	**0.000**	**0.000**	**0.000**	**0.000**	0.128	0.135	**0.000**	**0.000**	**0.000**	**0.000**	**0.000**	**0.000**	**0.000**	0.883	**0.000**	0.086	**0.047**	**0.000**	**0.000**
**Dif**	**N**	0.742	**0.000**		**0.000**	**0.000**	0.103	**0.000**	**0.000**	0.941	**0.000**	**0.000**	0.304	0.731	**0.000**	0.675	**0.000**	**0.000**	0.659	**0.000**	**0.000**	**0.000**	**0.000**	**0.000**	**0.000**
**Dif**	**E**	**0.000**	**0.000**	**0.000**		0.941	**0.000**	**0.000**	0.381	**0.000**	**0.000**	**0.000**	**0.000**	**0.000**	**0.000**	**0.000**	0.254	0.075	**0.000**	**0.000**	**0.000**	**0.000**	**0.000**	**0.000**	**0.000**
**Dif**	**U**	**0.000**	**0.000**	**0.000**	0.941		**0.000**	**0.000**	0.211	**0.000**	**0.000**	**0.000**	**0.000**	**0.000**	**0.000**	**0.000**	0.181	0.059	**0.000**	**0.000**	**0.000**	**0.000**	**0.000**	**0.000**	**0.000**
**Dif**	**F**	0.254	**0.000**	0.103	**0.000**	**0.000**		**0.000**	**0.000**	0.111	**0.000**	**0.000**	**0.001**	0.269	**0.000**	0.211	**0.000**	**0.000**	0.205	**0.000**	**0.000**	**0.000**	**0.000**	**0.000**	**0.000**
**Per**	**I**	**0.000**	**0.000**	**0.000**	**0.000**	**0.000**	**0.000**		**0.000**	**0.000**	0.115	0.107	**0.000**	**0.000**	0.393	**0.000**	**0.000**	**0.000**	**0.000**	**0.000**	0.341	0.192	0.141	0.825	0.454
**Per**	**O**	**0.000**	**0.000**	**0.000**	0.381	0.211	**0.000**	**0.000**		**0.000**	**0.000**	**0.000**	**0.000**	**0.000**	**0.000**	**0.000**	0.606	0.423	**0.000**	**0.000**	**0.000**	**0.000**	**0.000**	**0.000**	**0.000**
**Per**	**N**	0.850	**0.000**	0.941	**0.000**	**0.000**	0.111	**0.000**	**0.000**		**0.000**	**0.000**	0.162	0.804	**0.000**	0.728	**0.000**	**0.000**	0.864	**0.000**	**0.000**	**0.000**	**0.000**	**0.000**	**0.000**
**Per**	**E**	**0.000**	0.128	**0.000**	**0.000**	**0.000**	**0.000**	0.115	**0.000**	**0.000**		0.898	**0.000**	**0.000**	**0.003**	**0.000**	**0.000**	**0.000**	**0.000**	0.121	**0.006**	1.000	0.606	0.121	**0.007**
**Per**	**U**	**0.000**	0.135	**0.000**	**0.000**	**0.000**	**0.000**	0.107	**0.000**	**0.000**	0.898		**0.000**	**0.000**	**0.001**	**0.000**	**0.000**	**0.000**	**0.000**	0.077	**0.002**	0.659	0.774	0.085	**0.002**
**Per**	**F**	0.176	**0.000**	0.304	**0.000**	**0.000**	**0.001**	**0.000**	**0.000**	0.162	**0.000**	**0.000**		0.123	**0.006**	0.076	**0.000**	**0.000**	0.222	**0.000**	**0.011**	**0.000**	**0.000**	**0.000**	**0.007**
**Str**	**I**	0.768	**0.000**	0.731	**0.000**	**0.000**	0.269	**0.000**	**0.000**	0.804	**0.000**	**0.000**	0.123		**0.000**	0.768	**0.000**	**0.000**	0.826	**0.000**	**0.000**	**0.000**	**0.000**	**0.000**	**0.000**
**Str**	**O**	**0.000**	**0.000**	**0.000**	**0.000**	**0.000**	**0.000**	0.393	**0.000**	**0.000**	**0.003**	**0.001**	**0.006**	**0.000**		**0.000**	**0.000**	**0.000**	**0.000**	**0.000**	0.928	**0.004**	**0.015**	0.410	0.768
**Str**	**N**	0.826	**0.000**	0.675	**0.000**	**0.000**	0.211	**0.000**	**0.000**	0.728	**0.000**	**0.000**	0.076	0.768	**0.000**		**0.000**	**0.000**	0.813	**0.000**	**0.000**	**0.000**	**0.000**	**0.000**	**0.000**
**Str**	**E**	**0.000**	**0.000**	**0.000**	0.254	0.181	**0.000**	**0.000**	0.606	**0.000**	**0.000**	**0.000**	**0.000**	**0.000**	**0.000**	**0.000**		0.462	**0.000**	**0.000**	**0.000**	**0.000**	**0.000**	**0.000**	**0.000**
**Str**	**U**	**0.000**	**0.000**	**0.000**	0.075	0.059	**0.000**	**0.000**	0.423	**0.000**	**0.000**	**0.000**	**0.000**	**0.000**	**0.000**	**0.000**	0.462		**0.000**	**0.000**	**0.000**	**0.000**	**0.000**	**0.000**	**0.000**
**Str**	**F**	0.768	**0.000**	0.659	**0.000**	**0.000**	0.205	**0.000**	**0.000**	0.864	**0.000**	**0.000**	0.222	0.826	**0.000**	0.813	**0.000**	**0.000**		**0.000**	**0.000**	**0.000**	**0.000**	**0.000**	**0.000**
**Mot**	**I**	**0.000**	0.883	**0.000**	**0.000**	**0.000**	**0.000**	**0.000**	**0.000**	**0.000**	0.121	0.077	**0.000**	**0.000**	**0.000**	**0.000**	**0.000**	**0.000**	**0.000**		**0.000**	0.070	**0.050**	**0.000**	**0.000**
**Mot**	**O**	**0.000**	**0.000**	**0.000**	**0.000**	**0.000**	**0.000**	0.341	**0.000**	**0.000**	**0.006**	**0.002**	**0.011**	**0.000**	0.928	**0.000**	**0.000**	**0.000**	**0.000**	**0.000**		**0.009**	**0.021**	0.239	0.941
**Mot**	**N**	**0.000**	0.086	**0.000**	**0.000**	**0.000**	**0.000**	0.192	**0.000**	**0.000**	1.000	0.659	**0.000**	**0.000**	**0.004**	**0.000**	**0.000**	**0.000**	**0.000**	0.070	**0.009**		0.864	0.176	**0.010**
**Mot**	**E**	**0.000**	**0.047**	**0.000**	**0.000**	**0.000**	**0.000**	0.141	**0.000**	**0.000**	0.606	0.774	**0.000**	**0.000**	**0.015**	**0.000**	**0.000**	**0.000**	**0.000**	**0.050**	**0.021**	0.864		0.208	**0.027**
**Mot**	**U**	**0.000**	**0.000**	**0.000**	**0.000**	**0.000**	**0.000**	0.825	**0.000**	**0.000**	0.121	0.085	**0.000**	**0.000**	0.410	**0.000**	**0.000**	**0.000**	**0.000**	**0.000**	0.239	0.176	0.208		0.423
**Mot**	**F**	**0.000**	**0.000**	**0.000**	**0.000**	**0.000**	**0.000**	0.454	**0.000**	**0.000**	**0.007**	**0.002**	**0.007**	**0.000**	0.768	**0.000**	**0.000**	**0.000**	**0.000**	**0.000**	0.941	**0.010**	**0.027**	0.423

**Table:** (**b**)

**Quest.**		**Dif**	**Dif**	**Dif**	**Dif**	**Dif**	**Dif**	**Per**	**Per**	**Per**	**Per**	**Per**	**Per**	**Str**	**Str**	**Str**	**Str**	**Str**	**Str**	**Mot**	**Mot**	**Mot**	**Mot**	**Mot**	**Mot**
	**Seq.**	**I**	**O**	**N**	**E**	**U**	**F**	**I**	**O**	**N**	**E**	**U**	**F**	**I**	**O**	**N**	**E**	**U**	**F**	**I**	**O**	**N**	**E**	**U**	**F**
**Dif**	**I**		**0.000**	0.907	**0.000**	**0.000**	0.091	**0.000**	**0.000**	0.584	**0.000**	**0.000**	**0.011**	0.511	**0.000**	0.431	**0.000**	**0.000**	0.352	**0.000**	**0.000**	**0.000**	**0.000**	**0.000**	**0.000**
**Dif**	**O**	**0.000**		**0.000**	**0.000**	**0.000**	**0.000**	**0.000**	**0.000**	**0.000**	0.091	0.106	**0.000**	**0.000**	**0.000**	**0.000**	**0.000**	**0.000**	**0.000**	0.485	**0.000**	**0.043**	**0.030**	**0.000**	**0.000**
**Dif**	**N**	0.907	**0.000**		**0.000**	**0.000**	0.080	**0.000**	**0.000**	0.687	**0.000**	**0.000**	**0.012**	0.323	**0.000**	0.343	**0.000**	**0.000**	0.548	**0.000**	**0.000**	**0.000**	**0.000**	**0.000**	**0.000**
**Dif**	**E**	**0.000**	**0.000**	**0.000**		0.954	**0.000**	**0.000**	0.441	**0.000**	**0.000**	**0.000**	**0.000**	**0.000**	**0.000**	**0.000**	0.576	0.443	**0.000**	**0.000**	**0.000**	**0.000**	**0.000**	**0.000**	**0.000**
**Dif**	**U**	**0.000**	**0.000**	**0.000**	0.954		**0.000**	**0.000**	0.245	**0.000**	**0.000**	**0.000**	**0.000**	**0.000**	**0.000**	**0.000**	0.441	0.363	**0.000**	**0.000**	**0.000**	**0.000**	**0.000**	**0.000**	**0.000**
**Dif**	**F**	0.091	0.000	0.080	**0.000**	**0.000**		**0.000**	**0.000**	**0.009**	**0.000**	**0.000**	**0.000**	0.343	**0.000**	0.323	**0.000**	**0.000**	**0.008**	**0.000**	**0.000**	**0.000**	**0.000**	**0.000**	**0.000**
**Per**	**I**	**0.000**	**0.000**	**0.000**	**0.000**	**0.000**	**0.000**		**0.000**	**0.000**	**0.043**	**0.049**	**0.000**	**0.000**	**0.032**	**0.000**	**0.000**	**0.000**	**0.000**	**0.000**	0.375	0.091	0.155	0.771	0.181
**Per**	**O**	**0.000**	**0.000**	**0.000**	0.441	0.245	**0.000**	**0.000**		**0.000**	**0.000**	**0.000**	**0.000**	**0.000**	**0.000**	**0.000**	0.954	0.888	**0.000**	**0.000**	**0.000**	**0.000**	**0.000**	**0.000**	**0.000**
**Per**	**N**	0.584	**0.000**	0.687	**0.000**	**0.000**	**0.009**	**0.000**	**0.000**		**0.000**	**0.000**	**0.036**	0.223	**0.000**	0.124	**0.000**	**0.000**	0.954	**0.000**	**0.000**	**0.000**	**0.000**	**0.000**	**0.000**
**Per**	**E**	**0.000**	0.091	**0.000**	**0.000**	**0.000**	**0.000**	**0.043**	**0.000**	**0.000**		0.862	**0.000**	**0.000**	**0.000**	**0.000**	**0.000**	**0.000**	**0.000**	0.163	**0.001**	0.848	0.694	0.067	**0.000**
**Per**	**U**	**0.000**	0.106	**0.000**	**0.000**	**0.000**	**0.000**	**0.049**	**0.000**	**0.000**	0.862		**0.000**	**0.000**	**0.000**	**0.000**	**0.000**	**0.000**	**0.000**	0.259	**0.001**	0.798	0.523	0.069	**0.001**
**Per**	**F**	**0.011**	**0.000**	**0.012**	**0.000**	**0.000**	**0.000**	**0.000**	**0.000**	**0.036**	**0.000**	**0.000**		**0.000**	**0.027**	**0.000**	**0.000**	**0.000**	0.080	**0.000**	**0.001**	**0.000**	**0.000**	**0.000**	**0.002**
**Str**	**I**	0.511	**0.000**	0.323	**0.000**	**0.000**	0.343	**0.000**	**0.000**	0.223	**0.000**	**0.000**	**0.000**		**0.000**	0.684	**0.000**	**0.000**	0.175	**0.000**	**0.000**	**0.000**	**0.000**	**0.000**	**0.000**
**Str**	**O**	**0.000**	**0.000**	**0.000**	**0.000**	**0.000**	**0.000**	**0.032**	**0.000**	**0.000**	**0.000**	**0.000**	**0.027**	**0.000**		**0.000**	**0.000**	**0.000**	**0.000**	**0.000**	0.163	**0.000**	**0.000**	**0.021**	0.343
**Str**	**N**	0.431	**0.000**	0.343	**0.000**	**0.000**	0.323	**0.000**	**0.000**	0.124	**0.000**	**0.000**	**0.000**	0.684	**0.000**		**0.000**	**0.000**	0.104	**0.000**	**0.000**	**0.000**	**0.000**	**0.000**	**0.000**
**Str**	**E**	**0.000**	**0.000**	**0.000**	0.576	0.441	**0.000**	**0.000**	0.954	**0.000**	**0.000**	**0.000**	**0.000**	**0.000**	**0.000**	**0.000**		0.684	**0.000**	**0.000**	**0.000**	**0.000**	**0.000**	**0.000**	**0.000**
**Str**	**U**	**0.000**	**0.000**	**0.000**	0.443	0.363	**0.000**	**0.000**	0.888	**0.000**	**0.000**	**0.000**	**0.000**	**0.000**	**0.000**	**0.000**	0.684		**0.000**	**0.000**	**0.000**	**0.000**	**0.000**	**0.000**	**0.000**
**Str**	**F**	0.352	**0.000**	0.548	**0.000**	**0.000**	**0.008**	**0.000**	**0.000**	0.954	**0.000**	**0.000**	0.080	0.175	**0.000**	0.104	**0.000**	**0.000**		**0.000**	**0.000**	**0.000**	**0.000**	**0.000**	**0.000**
**Mot**	**I**	**0.000**	0.485	**0.000**	**0.000**	**0.000**	**0.000**	**0.000**	**0.000**	**0.000**	0.163	0.259	**0.000**	**0.000**	**0.000**	**0.000**	**0.000**	**0.000**	**0.000**		**0.000**	0.176	0.121	**0.001**	**0.000**
**Mot**	**O**	**0.000**	**0.000**	**0.000**	**0.000**	**0.000**	**0.000**	0.375	**0.000**	**0.000**	**0.001**	**0.001**	**0.001**	**0.000**	0.163	**0.000**	**0.000**	**0.000**	**0.000**	**0.000**		**0.005**	**0.008**	0.363	1.000
**Mot**	**N**	**0.000**	**0.043**	**0.000**	**0.000**	**0.000**	**0.000**	0.091	**0.000**	**0.000**	0.848	0.798	**0.000**	**0.000**	**0.000**	**0.000**	**0.000**	**0.000**	**0.000**	0.176	**0.005**		1.000	0.072	**0.003**
**Mot**	**E**	**0.000**	**0.030**	**0.000**	**0.000**	**0.000**	**0.000**	0.155	**0.000**	**0.000**	0.694	0.523	**0.000**	**0.000**	**0.000**	**0.000**	**0.000**	**0.000**	**0.000**	0.121	**0.008**	1.000		0.169	**0.005**
**Mot**	**U**	**0.000**	**0.000**	**0.000**	**0.000**	**0.000**	**0.000**	0.771	**0.000**	**0.000**	0.067	0.069	**0.000**	**0.000**	**0.021**	**0.000**	**0.000**	**0.000**	**0.000**	**0.001**	0.363	0.072	0.169		0.234
**Mot**	**F**	**0.000**	**0.000**	**0.000**	**0.000**	**0.000**	**0.000**	0.181	**0.000**	**0.000**	**0.000**	**0.001**	**0.002**	**0.000**	0.343	**0.000**	**0.000**	**0.000**	**0.000**	**0.000**	1.000	**0.003**	**0.005**	0.234	
